# The Tomato Transcription Factor RAV Affects the Systemic Infection of TYLCV by Interacting With V2

**DOI:** 10.1111/mpp.70230

**Published:** 2026-02-23

**Authors:** Chenwei Zhang, Xin Jia, Guiyan Fan, Xiaoli Ren, Xing Han, Xiaocong Jiao, Yuan Cheng, Yajuan Cheng, Zihan Liu, Zhehao Yuan, Yongmin Chen

**Affiliations:** ^1^ Department of Agriculture and Food Science, Modern Agricultural Science and Technology Laboratory Shijiazhuang University Shijiazhuang China

**Keywords:** plant antiviral pathways, protein interaction, SlRAV2, TYLCV, V2

## Abstract

Tomato yellow leaf curl virus (TYLCV) is a widely distributed pathogen responsible for tomato yellow leaf curl disease. However, the molecular mechanism underlying TYLCV infection and the plant antivirus pathway remains unclear. This study reports that tomato ( *Solanum lycopersicum* ) RELATED TO ABSCISIC ACID INSENSITIVE3/VIVIPAROUS2 (SlRAV2), a transcription factor of the APETALA2/ETHYLENE RESPONSE FACTOR (AP2/ERF) superfamily containing an AP2/B3 DNA‐binding domain, suppresses systemic TYLCV accumulation by interacting with the V2 protein, an RNA silencing suppressor (RSS) encoded by TYLCV. Transient overexpression and silencing of SlRAV2 altered TYLCV levels in systemic leaves, prompting us to investigate the underlying mechanism. Molecular analyses revealed that SlRAV2 directly binds to the promoter of the pathogenesis‐related protein 1 (*SlPR1*) gene, thereby activating its expression and contributing to basal defence responses. V2 affects the nuclear import of SlRAV2 through protein–protein interaction and recruits SlRAV2 to bind more 21‐nt double‐stranded siRNA, thereby enhancing its own RSS activity and transiently increasing local TYLCV accumulation. Moreover, nuclear‐localised V2 increases SlRAV2 binding affinity for the *SlPR1* promoter, resulting in elevated *SlPR1* transcription and concomitant accumulation of reactive oxygen species (ROS). Our findings reveal a novel antiviral mechanism whereby SlRAV2 interacts with V2 to enhance the local accumulation of TYLCV transiently, causing localised cell necrosis and preventing viral spread from infiltrated leaves to systemic leaves, thereby suppressing the systemic infection of TYLCV.

## Graphical abstract

This study investigated the interaction between TYLCV V2 and tomato transcription factor SlRAV2: SlRAV2 enhances the RSS activity of V2 by strengthening its binding to 21‐nt ds‐siRNA, V2 enhances SlRAV2's *SlPR1* promoter regulation and their interaction inhibits systemic spread of the virus from infiltrated leaves to systemic leaves.

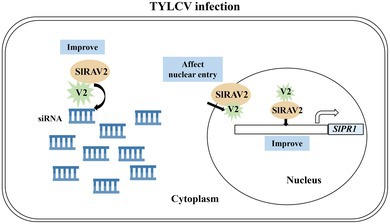

## 1 Introduction

Tomato cultivation is plagued by a spectrum of biological stressors, including viral infections, which profoundly impede the growth and severely undermine tomato yield and quality (Wu et al. 2006; Li et al. 2022; Cao et al. 2024). Among these, tomato yellow leaf curl virus (TYLCV)—a member of the *Geminiviridae* family and *Begomovirus* genus—is one of the most economically destructive pathogens affecting tomato crops worldwide (Moriones and Navas‐Castillo 2000). TYLCV has a broad host range and exhibits high transmission efficiency, primarily via the whitefly vector *Bemisia tabaci* (*Hemiptera: Aleyrodidae*) through a persistent circulative mode of transmission (Goldman and Czosnek 2002). Environmental stressors such as drought and extreme temperatures exacerbate disease severity, further compounding yield losses (Yue et al. 2024). TYLCV possesses a circular single‐stranded DNA genome whose open reading frames (ORFs) encode proteins that contribute to virus replication, movement, and suppression of host defence mechanisms. The viral sense‐strand encodes the coat protein (V1) and V2 protein, while the complementary‐sense strand has six additional ORFs (C1, C2, C3, C4, C5, C6) and a recently identified C7 (Gorovits et al. 2017; Zhao et al. 2018; Liu et al. 2023; Zhao et al. 2023). V2 functions as an RNA silencing suppressor (RSS) protein capable of binding to double‐stranded small interfering RNAs (ds siRNAs) to prevent both transcriptional gene silencing (TGS) and post‐transcriptional gene silencing (PTGS) (Wartig et al. 1997; Rojas et al. 2001; Fukunaga and Doudna 2009; Li et al. 2014; Zrachya et al. 2007; Luna et al. 2012) by disrupting the host RNA interference (RNAi) machinery. Notably, a single amino acid residue of V2 has been shown to mediate its self‐interaction, aggregate formation and pathogenicity of TYLCV (Zhao et al. 2018).

V2 exerts its effects through several distinct mechanisms. It interacts with the key components of the RNA silencing complex (RISC), including proteins Argonaute 4 (AGO4) and Suppressor of Gene Silencing 3 (SGS3) in the RNA silencing pathway, thereby influencing the assembly of the RISC and compromising the antiviral RNA silencing process (Glick et al. 2018). Additionally, V2 interacts with host histone deacetylase 6 to suppress methylation‐mediated transcriptional gene silencing (Wang et al. 2018). It also modulates the activity of CYP1, a papain‐like cysteine protease implicated in plant immune signalling, thereby influencing downstream defence responses (Bar‐Ziv et al. 2015, 2012). Furthermore, V2 cooperates with another TYLCV‐encoded protein, C4, to enhance its accumulation independently of V2 RNA silencing suppression activity (Wang et al. 2022). Beyond its role in pathogenesis, V2 can also facilitate the nuclear export of V1 (CP) encoded by TYLCV (Zhao et al. 2020). Viral proteins frequently engage in interactions with host proteins to enhance the level of infection and suppress plant immunity. Besides V2, C4 also functions as an RSS that inhibits systemic silencing and has been shown to interact with host proteins involved in RNA silencing, translation, ubiquitination, pathogenesis‐related processes, and plant defence responses (Garnelo Gómez et al. 2019; Kim et al. 2016). Accumulating evidence suggests that many RSSs from different plant viruses often interact or target plant antiviral proteins, especially certain transcription factors (TFs) related to disease resistance pathways (Poque et al. 2018; Demi̇rel et al. 2025; Li et al. 2014; Wu et al. 2019). However, the specific functions, functional diversity, and underlying molecular mechanisms of these TFs in mediating antiviral responses remain largely elusive. Elucidating these interactions represents a critical step toward understanding plant–virus coevolution and developing novel strategies for durable disease resistance.

The APETALA2/ETHYLENE RESPONSE FACTOR (AP2/ERF) superfamily of TFs is classified into three separate subfamilies—AP2, RAV (related to ABI3/VP1), and ERF—based on the number of AP2 DNA‐binding domains and sequence similarity (Licausi et al. 2013). Among them, the RAV protein contains AP2 and B3 domains (Yamasaki 2004). Previous studies have demonstrated that RAVs are involved in various aspects of plant development, including axillary bud outgrowth, floral development regulation, and photoperiod regulation (Kagaya et al. 1999; Zhao, Liu, et al. 2021; Sohn et al. 2006; Li et al. 2011; Pei et al. 2024; Zhu et al. 2025; Woo et al. 2010; Kim et al. 2005; Cheng et al. 2023; Xue et al. 2025). Emerging evidence has highlighted the complex regulatory roles of RAV TFs in plants' biotic stress responses (Pre et al. 2008). More recently, RAVs have also been implicated in salicylic acid (SA)‐mediated defence responses (Zhang et al. 2022; Xie et al. 2024; Zhang, Jia, et al. 2025). For instance, the silencing suppressor HC‐Pro encoded by tobacco etch virus (TEV) and p38 encoded by turnip crinkle virus (TCV) can utilise host AtRAV2 to inhibit the host antiviral RNA silencing pathways (Endres et al. 2010). In grapevine, VvRAV1 interacts with p24 encoded by grapevine leafroll‐associated virus 2 (GLRaV2), and this interaction modulates VvRAV1‐mediated regulation of the defence gene *VvPR1* (Zhang et al. 2022). Similarly, in pear, PbRAV1 can enhance resistance to apple stem grooving virus (ASGV) by directly interacting with the RSS (CP) encoded by ASGV (Xie et al. 2024). In rice, OsRAV2 interacts with LHCB5, a protein associated with *Magnaporthe oryzae* resistance, regulates proline, malondialdehyde, hydrogen peroxide and abscisic acid (ABA)‐signalling under drought stress, and enhances resistance to rice powdery mildew by increasing the expression of genes related to disease resistance (Fu et al. 2025). Furthermore, OsRAV15 can interact with OsMYC2, a key integrator of jasmonic acid (JA) signalling, to activate the JA‐mediated disease defence response pathway (Zhang, Huang, et al. 2025). In addition to RAV, other plant proteins associated with disease resistance have also been shown to target viral proteins. For example, WRKY40 modulates tomato mosaic virus (ToMV) infection by altering viral protein levels (Jiang et al. 2021). SlGRXC6 interacts with V2 of TYLCV to inhibit its nuclear export and affect virus infection of the plant (Zhao, Zhou, et al. 2021). HYD1 in cucumber recognises the P1b protein encoded by cucumber vein yellowing virus (CVYV), ultimately affecting CVYV replication (García et al. 2021). Therefore, interactions between TFs and viral proteins constitute a prevalent mechanism by which plants modulate viral infection.

Previous research results have demonstrated that successful virus infection depends on an interaction between the plant and the viral factors as well as the plant's intrinsic defence response (Tan et al. 2024). However, the mechanisms governing viral invasion and specific host factors involved remain incompletely understood. To gain deeper insights into the host–virus interaction network, a TYLCV infection was employed in our previous study, which revealed that TYLCV infection significantly increases the expression of plant genes associated with disease resistance (Zhang, Jia, et al. 2025). Building upon these findings, the present study employed the viral protein V2 as the bait in a yeast two‐hybrid (Y2H) screen of a tomato cDNA library, leading to the identification of an interacting protein belonging to the AP2/ERF family and RAV subfamily. Here, we demonstrate that TYLCV protein V2 interacts with the tomato antiviral protein SlRAV2 (Solyc10g086270.ITAG4.0, GenBank: NM_001320461.2), with reciprocal impacts on their respective functions. Our results reveal a molecular mechanism underlying the role of SlRAV2 in resisting TYLCV systemic infection: SlRAV2 can inhibit the systemic spread of TYLCV by interacting with V2 and influencing its RSS activity, and SlRAV2 also exploits this interaction to enhance its regulation effect on the gene *SlPR1*, a hallmark gene of the SA‐mediated defence response. These findings provide novel insights into the host–virus interaction landscape and offer valuable guidance for the development of effective strategies to control the infection of TYLCV.

## 2 Results

### 2.1 Tomato Transcription Factor SlRAV2 Interacts With TYLCV V2

**FIGURE 1:**
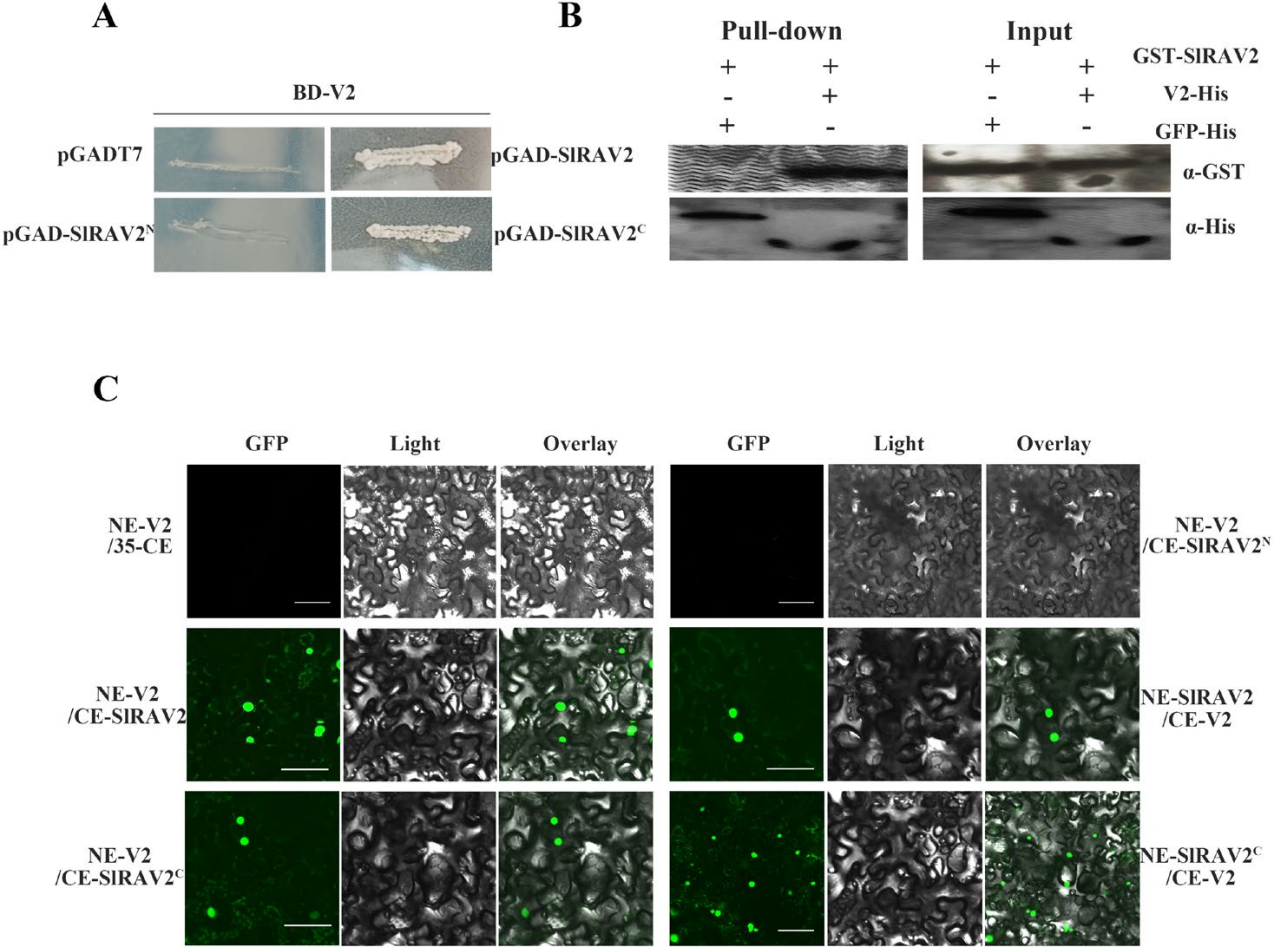
Interaction between V2 and SlRAV2. (A) Interactions between V2 and SlRAV2 in yeast. Yeast strain AH109 co‐transformed with plasmids expressing the fusion proteins were plated on SD/−Leu/−Trp/−His/−Ade. Transformation of pGADT7 and pGBK‐V2 served as negative controls. (B) In vitro pull‐down to determine the interaction between V2 and SlRAV2. The combination GFP‐His/GST‐SlRAV2 served as the negative control. Both the input and pull‐down samples were detected by western blotting with anti‐His and anti‐GST antibodies. (C) In vivo bimolecular fluorescence complementation of V2/SlRAV2 interactions. The proteins were expressed in *Nicotiana benthamiana* leaves. Co‐expression of V2‐YFP^N^ and 35S‐YFP^C^ served as the negative control. Confocal micrographs were taken 3 days post‐infiltration. Bars = 50 μm. Each assay was repeated three times.

Previously, using the V2 (GenBank: PP737594) protein encoded by TYLCV as bait, Y2H screening identified an interacting clone encoding a peptide of the C terminus of SlRAV2 from the tomato cDNA library. The sequence analysis revealed that *SlRAV2* encodes a protein of 395 amino acids (aa) containing AP2 and B3 domains. The full sequences of *SlRAV2* and *V2* were cloned into pGADT7 and pGBKT7 vectors, respectively, and co‐transformed into yeast competent cells. Yeast cells co‐expressing SlRAV2 and V2 grew on the synthetic drop‐out medium lacking Trp, Leu, His and Ade (SD/−Leu/−Trp/−His/−Ade) medium, whereas no growth was observed for the negative control (pGBK‐V2/pGAD‐T7) (Figure 1A). To map the interacting region, SlRAV2 was divided into two segments: the N‐terminal region containing the AP2 domain (SlRAV2^N^, 1–150 aa) and the C‐terminal region containing the B3 domain (SlRAV2^C^, 151–395 aa). Y2H assays revealed that SlRAV2^C^ but not SlRAV2^N^ interacted with V2 (Figure 1A). This interaction was further validated in vitro by pull‐down assays, in which GST‐SlRAV2 interacted with V2‐His (Figure 1B), whereas no interaction was detected between GST‐SlRAV2 and the unrelated protein GFP‐His (Figure 1B, lane without band). A bimolecular fluorescence complementation (BiFC) assay in planta further supported the SlRAV2/V2 and SlRAV2^C^/V2 interactions, yielding results consistent with the Y2H and pull‐down experiments (Figure 1C). These results suggest that following TYLCV infection, V2 physically interacts with the plant transcription factor SlRAV2, raising the possibility that SlRAV2, as a potential antiviral protein, may interact with the protein of the virus to resist viral infection. However, the functional relevance of this interaction remains to be elucidated.

### 2.2 The Effect of SlRAV2 on TYLCV Infection

**FIGURE 2:**
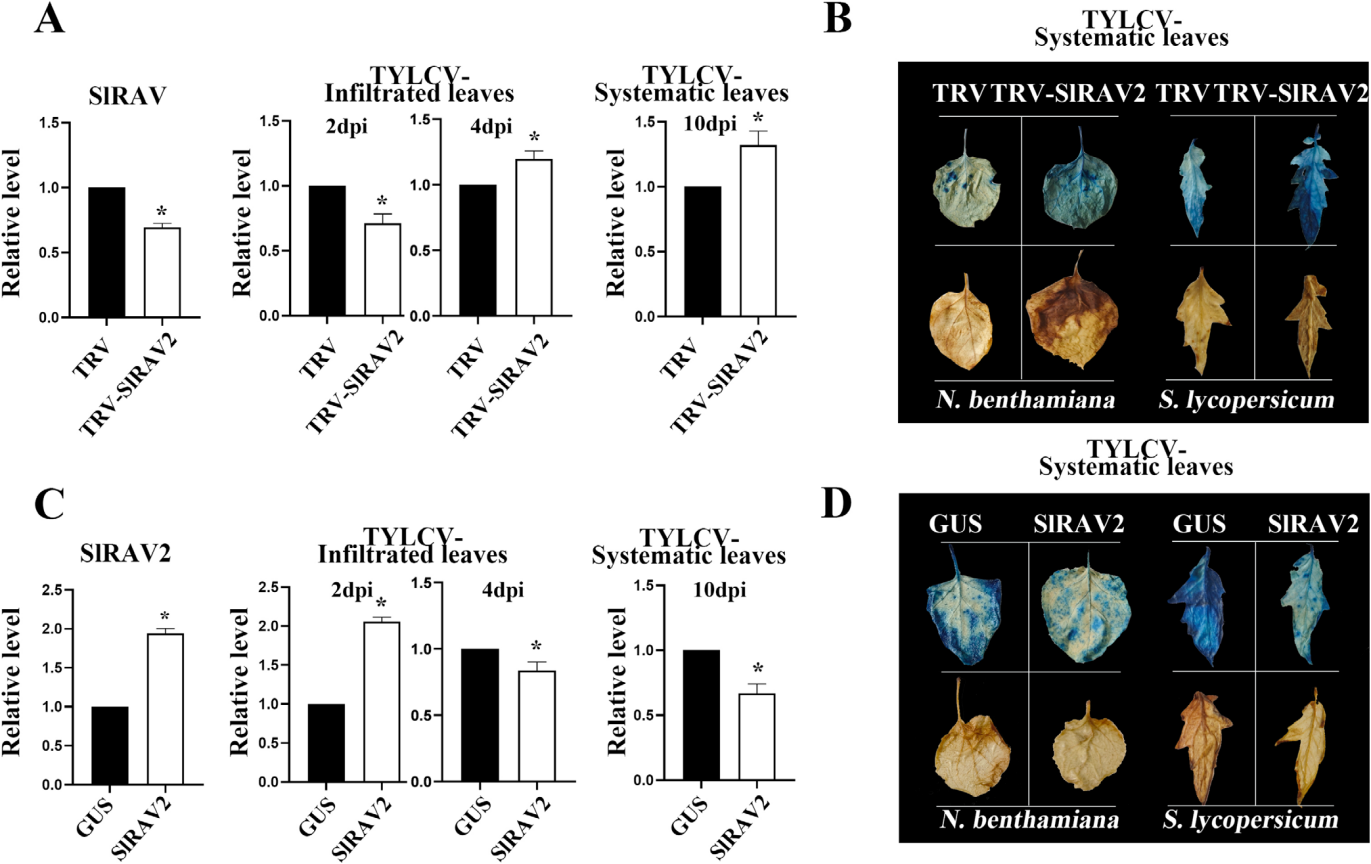
Modulation of SlRAV2 expression levels influences tomato yellow leaf curl virus (TYLCV) DNA accumulation and symptom development. (A) Silencing *SlRAV2* increased TYLCV accumulation in tomato systemic leaves. Tomato plants were subjected to virus inoculation 3 days post‐*SlRAV2* silencing. Viral DNA levels in both infiltrated and systemic leaves were quantified by quantitative PCR (qPCR) at 2, 4, 10 days post‐inoculation (dpi). Data are presented as relative expression levels compared to the control. qPCR results are shown as mean ± SD of three independent experiments. SD is denoted by error bars. (B) *SlRAV2* silencing enhanced staining intensity in tomato systemic leaves (right panel). Staining of systemic leaves with 3,3′‐diaminobenzidine (DAB) and trypan blue after *SlRAV2* silencing was more intense than the negative control. Similar results were observed following *SlRAV2* silencing in *Nicotiana benthamiana* (left panel). (C) Overexpression *SlRAV2* in tomato reduced TYLCV accumulation in systemic leaves. Tomato plants were subjected to virus inoculation 3 days post‐*SlRAV2* overexpression. Expression levels of *SlRAV2* and TYLCV were measured in both infiltrated and systemic leaves by qPCR. Data are presented as relative expression levels. (D) *SlRAV2* overexpression reduced the DAB and trypan blue staining intensity in tomato systemic leaves (right panel). Similar results were observed following *SlRAV2* overexpression in *N. benthamiana* (left panel). Significant difference, *p < 0.05.

Previous experimental findings have shown that *SlRAV2* expression undergoes significant alterations following viral infection (Zhang, Jia, et al. 2025). Transient gene silencing and overexpression of *SlRAV2* in tomato plants revealed contrasting effects on TYLCV accumulation over time. Silencing *SlRAV2* resulted in reduced viral accumulation in the infiltrated leaves at 2 days post‐infiltration (dpi); however, viral accumulation increased by 4 dpi (Figure 2A). By 10 dpi, viral accumulation in the systemic leaves exceeded that of the negative control (Figure 2A). Conversely, pre‐inoculation overexpression of *SlRAV2* led to elevated TYLCV DNA accumulation in the infiltrated leaves at 2 dpi, followed by a decline at 4 dpi (Figure 2C). At 10 dpi, viral accumulation in systemic leaves was lower than that observed in the negative control (Figure 2C). Collectively, these experiments suggest that *SlRAV2* expression modulates viral accumulation in both infiltrated and systemic leaves. Histochemical analysis using trypan blue and 3,3′‐diaminobenzidine (DAB) staining revealed that silencing *SlRAV2* enhanced systemic leaf necrosis and elevated reactive oxygen (ROS) levels relative to the control group (Figure 2B). In contrast, transient overexpression of *SlRAV2* attenuated TYLCV‐induced necrosis in systemic leaves and reduced ROS accumulation compared with negative control (Figure 2D). Consistent staining outcomes were observed in *Nicotiana benthamiana* plants (Figure 2B,D). Staining results and corresponding analyses for tomato and *N. benthamiana* infiltrated leaves are provided in the Figure S1. Given that *SlRAV2* overexpression and silencing alter TYLCV levels in systemic and infiltrated leaves of tomato and *N. benthamiana*, we propose that SlRAV2 may confer resistance to TYLCV by enhancing localised necrosis responses at the infection site, thereby restricting viral spread to the systemic leaves.

### 2.3 SlRAV2 Enhances the Ability of V2 to Suppress RNA Silencing

**FIGURE 3:**
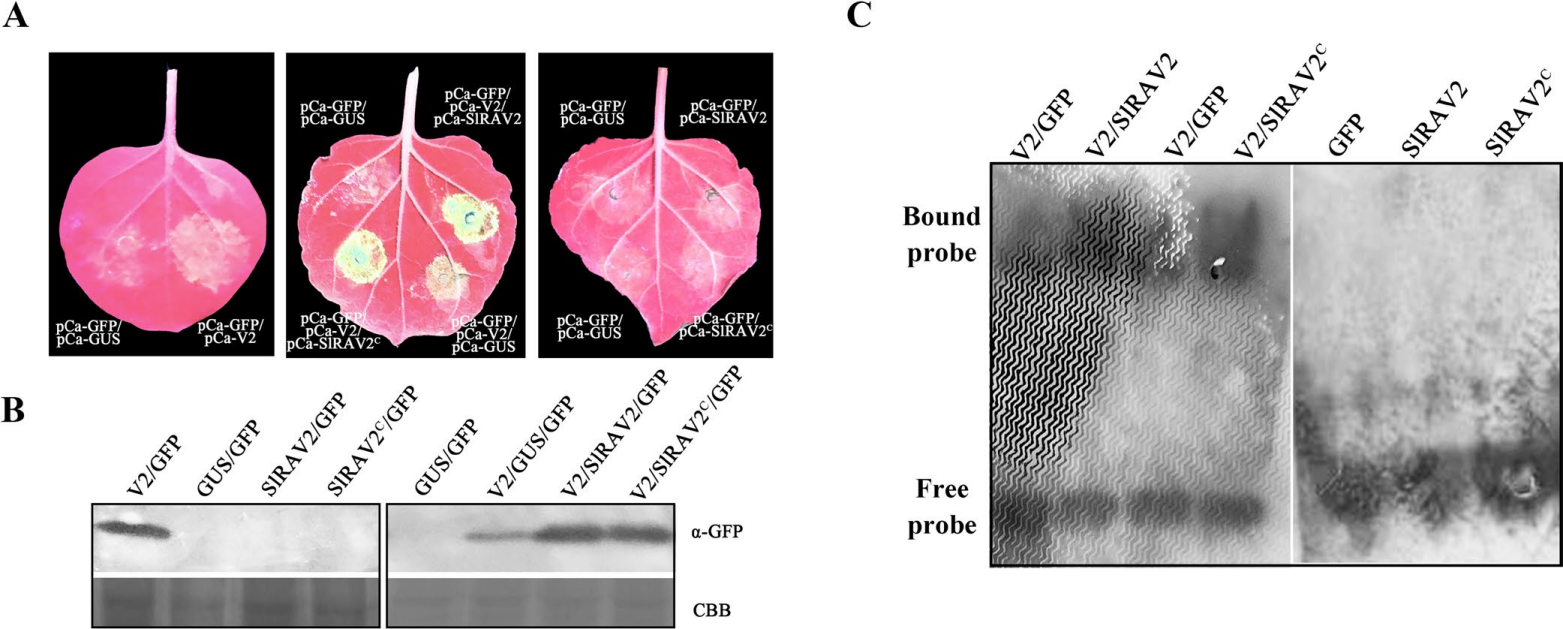
SlRAV2 enhanced the silencing suppressor activity of V2 by enhancing its ability of binding double‐stranded (ds) small interfering RNAs (siRNAs). (A) SlRAV2 enhanced the RNA silencing suppressor (RSS) activity of V2. The pCambia‐V2/pCambia‐GFP/pCambia‐SlRAV2 was transiently expressed in *Nicotiana benthamiana* by *Agrobacterium*‐mediated transient infection. The GFP fluorescence was observed under a UV light and photographed 3 days post‐infiltration. (B) Western blot results. Total protein was extracted from the GFP fluorescent area and subjected to western blot analysis with anti‐GFP antibodies. Coomassie Brilliant Blue (CBB) staining was used for protein detection to verify equal protein loading. (C) Electrophoretic mobility shift assay (EMSA) results. EMSA showed that SlRAV2 enhanced the ability of V2 to bind to 21‐nucleotide (nt) ds siRNA. GST‐SlRAV2 and V2‐His were expressed in prokaryotes and incubated with biotin‐labelled 21‐nt ds siRNA for EMSA. The addition of GST‐SlRAV2 protein enhanced the ability of V2 to bind to 21‐nt ds siRNA. GFP‐His served as a negative control.

SlRAV2 interacts directly with V2, and its expression level influences TYLCV accumulation, suggesting that SlRAV2 may modulate TYLCV infection by regulating the function of V2. To investigate this hypothesis, we performed transient co‐expression assays in *N. benthamiana* using various plasmid combinations. Under ultraviolet (UV) light, co‐expression of pCambia‐V2 and pCambia‐GFP maintained green fluorescence, while the negative control group (pCambia‐GUS/pCambia‐GFP) showed no obvious fluorescence (Figure 3A, left panel). Notably, co‐infiltration of pCambia‐V2/pCambia‐GFP/pCambia‐SlRAV2 or pCambia‐V2/pCambia‐GFP/pCambia‐SlRAV2^C^ led to enhanced fluorescence intensity compared to the negative control group expressing pCambia‐V2/pCambia‐GFP/pCambia‐GUS at 3 dpi (Figure 3A, middle panel). Western blot analysis confirmed that co‐expression of SlRAV2/V2 or SlRAV2^C^/V2 elevated GFP expression levels (Figure 3B, right panel), further supporting the conclusion that SlRAV2 enhanced the ability of V2 to suppress GFP silencing (Figure 3A,B). Additionally, neither pCambia‐SlRAV2/pCambia‐GFP nor pCambia‐SlRAV2^C^/pCambia‐GFP produced detectable fluorescence signals, and western blot analysis confirmed the absence of GFP expression (Figure 3A, right panel; Figure 3B left panel), indicating that SlRAV2 itself does not possess RSS activity.

Mechanistic investigations have established that most RSSs inhibit RNA silencing by binding to ds siRNA. Consistent with this model, V2 may also exert its RSS function through a similar mechanism. Using recombinant GST‐SlRAV2 and V2‐His fusion proteins (Figure S2) and biotin‐labelled 21‐nucleotide (nt) ds siRNA probes, electrophoretic mobility shift assay (EMSA) demonstrated that V2 binds to 21‐nt ds siRNA, supporting its role as an RSS (Figure 3C). Given the interaction between SlRAV2 and V2, and the observed modulation of V2's silencing suppression activity, we hypothesised that SlRAV2 may influence this activity by affecting V2's ds siRNA binding capacity. Indeed, EMSA results showed the presence of GST‐SlRAV2 or GST‐SlRAV2^C^ significantly enhanced the binding affinity of V2‐His for 21‐nt ds siRNA (Figure 3C). The EMSA results support the observations in Figure 3A,B, and demonstrate that SlRAV2 enhances the RNA silencing activity of V2 via interaction, specifically through modulation of its ds siRNA‐binding capability.

### 2.4 V2 Enhances SlRAV2‐Mediated Transcription Activation of *SlPR1*

**FIGURE 4:**
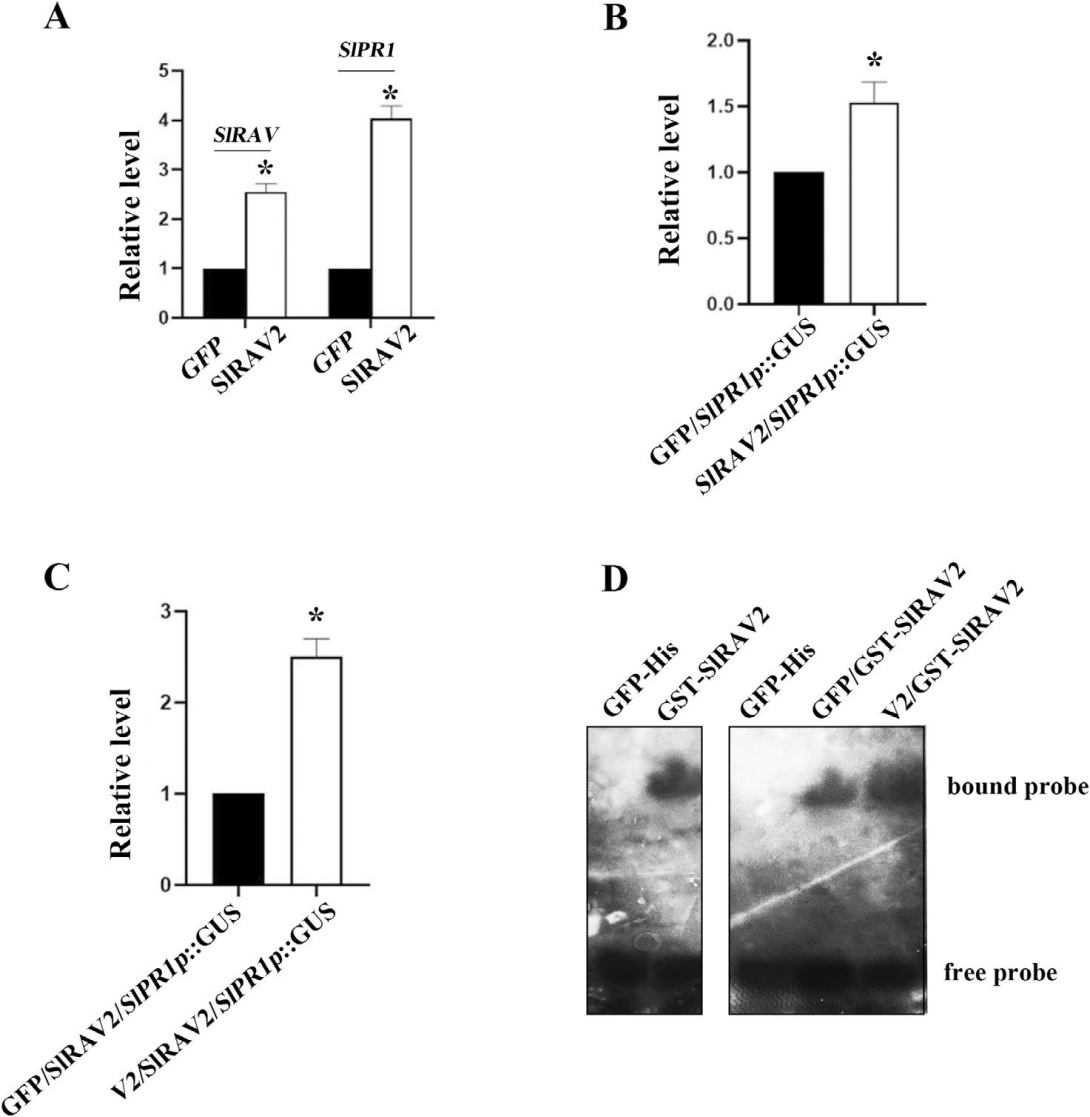
V2 affects the regulation of the *SlPR1* gene by interaction with SlRAV2. (A) SlRAV2 has a positive regulatory effect on *SlPR1*. SlRAV2 was overexpressed using the *Agrobacterium*‐mediated transient infection technology. The expression of *SlPR1* was detected using reverse transcription‐quantitative PCR. ‘*’ indicates a significant difference, *p* < 0.05. (B) SlRAV2 activates the *SlPR1* promoter. Leaf tissue from each treatment was harvested at 3 days post‐infiltration, and pooled samples were used for protein extraction and subsequent β‐glucuronidase (GUS) activity assays with 4‐methylumbelliferyl β‐D‐glucuronide (MUG) as the substrate. (C) V2 affects SlRAV2's function. Co‐expression of V2 with SlRAV2 enhanced the SlRAV2‐induced activation of the *SlPR1* promoter, as evidenced by a significant increase in GUS activity. ‘*’ indicates a significant difference, *p* < 0.05. (D) V2 enhances binding affinity of SlRAV2 for the ‘CACCTG’ motif in the *SlPR1* promoter. GST‐SlRAV2 and V2‐His protein were incubated with a DNA probe for the electrophoretic mobility shift assay. GFP‐His served as a control.

The interaction between SlRAV2 and V2 does not solely affect the function of V2. Previous studies have suggested that the members of the RAV TF family may participate in SA‐mediated defence response (Zhang et al. 2022). Consistent with this notion, overexpression of *SlRAV2* resulted in a 1.4‐fold increase in its own transcript level and a 2.9‐fold increase of *SlPR1* expression (Figure 4A). To further investigate whether SlRAV2 regulates *SlPR1* at the transcriptional level, we cloned the 2000‐bp promoter region of the *SlPR1* gene into the pBI121 vector, placing it upstream of the *GUS* reporter gene (designated *SlPR1*::GUS). Transient expression assays coupled with a promoter transactivation assay (based on GUS activity quantification) revealed that SlRAV2 enhanced GUS expression compared to the negative control expressing unrelated proteins (Figure 4B). This result suggests that SlRAV2 regulates *SlPR1* expression, possibly through direct modulation of its promoter (*SlPR1*p). Notably, co‐expression of V2 with SlRAV2 further elevated GUS activity to 230% relative to the negative control group (Figure 4C), implying that SlRAV2 may employ V2 to enhance transcriptional activation of *SlPR1*. EMSA results provided mechanistic insight into this regulation: GST‐SlRAV2 directly bound to a DNA probe containing a conserved ‘CACCTG’ motif derived from *SlPR1p*. Moreover, the presence of V2‐His enhanced the formation of the GST‐SlRAV2 binding to the *SlPR1*p (Figure 4D). Taken together, these results indicate that SlRAV2 may utilise V2 to augment its binding affinity to the *SlPR1*p, thereby reinforcing *SlPR1* expression. Given that *SlPR1* is a key downstream effector of the SA‐mediated defence response against TYLCV, our results provide indirect evidence that SlRAV2 suppresses systemic TYLCV accumulation, potentially by activating the SA signalling pathway, which in turn upregulates *SlPR1* expression to mount an effective defence.

### 2.5 V2 Affects the Subcellular Localisation of SlRAV2

**FIGURE 5:**
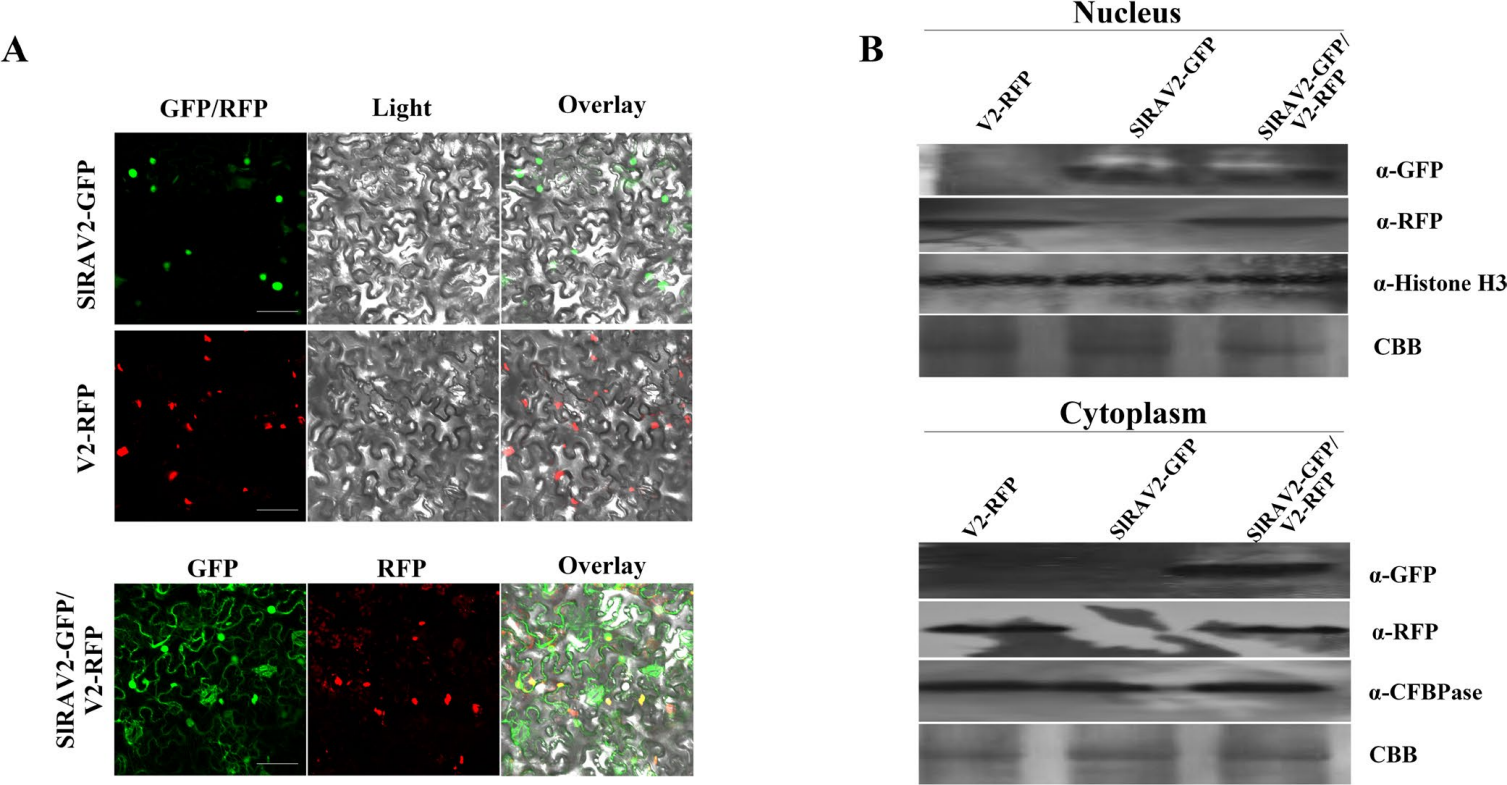
The V2–SlRAV2 interaction alters the subcellular localisation of SlRAV2. (A) The subcellular localisation of V2 and SlRAV2. Confocal micrographs of *Nicotiana benthamiana* epidermal cells at 3 days post‐infiltration (dpi). SlRAV2‐GFP localises exclusively to the nucleus (top row). V2‐RFP localises both in nucleus and cytoplasmic (middle row). Co‐expression of both proteins leads to the nuclear and cytoplasmic localisation of each. Scale bar = 50 μm. (B) Detection of V2 and SlRAV2 in nuclear and cytoplasmic fractions. Agroinfiltrated tissues of each treatment were sampled 3 dpi and pooled for protein extraction. Histone H3 and cytosolic fructose‐1,6‐bisphosphatase (cFBPase) served as markers for nuclear and cytoplasmic fractions, respectively, validating the purity of the isolation. Coomassie Brilliant Blue (CBB) staining verified equal protein loading. Upper, nuclear fraction; lower, cytoplasmic fraction. Leaves of *N. benthamiana* plants expressing the indicated protein(s), and confocal micrographs were taken 3 dpi. Bars = 50 μm.

Because SlRAV2 interacts with V2 and reciprocally influences their respective functions, we hypothesised that their subcellular localisation patterns might likewise be interdependent. Confocal microscope showed that V2 predominantly localised to both the nucleus and cytoplasm, exhibiting granular aggregates within the cytoplasm compartment (Figure 5A). In contrast, SlRAV2 was exclusively localised to the nucleus (Figure 5A). Upon co‐expression in *N. benthamiana* leaves, however, SlRAV2 localisation shifted significantly—from exclusive nuclear localisation to concurrent nuclear and cytoplasmic distribution—while V2 maintained its original localisation pattern (Figure 5A). To confirm these observations from the perspective of proteins, nuclear‐cytoplasmic fractionation assays were conducted. Consistent with confocal imaging, western blot analysis of isolated fractions demonstrated that both V2‐RFP and GFP‐SlRAV2 were present in the nucleus and cytoplasm when co‐expressed (Figure 5B). The above experiments confirmed that V2 alters the subcellular localisation of SlRAV2, probably facilitating its translocation into the cytoplasm or affecting its translocation into the nucleus.

The observed co‐localisation of V2 and SlRAV2 suggested that V2 may interfere with the nuclear import of SlRAV2, potentially through modulation of interaction between SlRAV2 and Importin α—a key nuclear transport receptor responsible for mediating the nuclear import of proteins harbouring nuclear localisation signals (NLSs). To test this hypothesis, we performed a Y2H assay using pGAD‐SlRAV2 and pGBK‐SlIMP (Importin α). The results showed a direct interaction between SlRAV2 and Importin α (Figure S3). Interestingly, in an in vitro pull‐down assay, the addition of V2‐His diminished the interaction between GST‐SlRAV2 and MBP‐SlIMP (Figure S3), suggesting that V2 competes with Importin α for binding to SlRAV2. Collectively, these findings indicate that the interaction between SlRAV2 and V2 influences SlRAV2's subcellular localisation and modulates its functional activity—probably by interfering with its nuclear import machinery.

## 3 Discussion

TYLCV is a widespread pathogen responsible for tomato yellow leaf curl disease, leading to substantial reductions in tomato yields. Given that plant antiviral proteins can suppress viral infection through direct interactions with the viral‐encoded proteins, we report the functional relationship between the tomato transcription factor SlRAV2 and the V2 protein encoded by TYLCV. This interaction enhanced TYLCV accumulation transiently in the infiltrated leaves, accompanied by an increase in the degree of cell necrosis; subsequently, it decreased local viral levels and inhibited the spread of TYLCV to the systemic leaves.

Previous studies using a TYLCV infectious clone experiments identified marked alterations in disease resistance‐related genes in both tomato and *N. benthamiana* plants. Among them, one *RAV* gene (Solyc10g086270.ITAG4.0) was significantly upregulated in TYLCV‐infected tomato plants (Zhang, Jia, et al. 2025). Based on these observations, we hypothesised that modulation of *SlRAV2* expression might influence TYLCV accumulation. Indeed, transient overexpression of *SlRAV2* in the TYLCV‐infected leaves initially led to a temporary increase in viral accumulation in the infiltrated leaves, followed by a reduction in viral levels in the infiltrated leaves and inhibiting the spread of TYLCV to the systemic leaves (Figure 2B,D). Conversely, silencing *SlRAV2* resulted in more extensive systemic infection (Figure 2A,C). Thus, these findings suggest that SlRAV2 may contribute to the early plant response to TYLCV, although its precise role requires further validation using stable transgenic approaches. Overexpressing *SlRAV2* also led to elevated ROS content and increased the number of necrotic spots in the infiltrated leaves, thereby inhibiting the systemic TYLCV spread. Similar phenomena have been reported in previous studies, wherein modulation of ROS levels enhance plant resistance to viruses (Hernández et al. 2016; Kozieł et al. 2024; Raza et al. 2021; Su et al. 2021; Hong et al. 2005). Changing the ROS content in plants can lead to a rapid outbreak of viruses within the plants, resulting in inhibition of the systemic infection of viruses (Király et al. 2025; Luo et al. 2023). Consistent with these reports, our finding suggests that SlRAV2 indirectly enhanced the hypersensitive response (HR), resulting in a local increase in the ROS content and initiation of the plant defence response, thereby inhibiting virus spread. Furthermore, DAB and trypan blue staining were less visually striking in infiltrated leaves compared to systemic leaves (Figure 2B,D). At 2 dpi, no significant differences in staining intensity were observed between the experimental groups and the control; by 4 dpi, leaves overexpressing *SlRAV2* exhibited slightly intensified DAB and trypan blue staining, whereas *SlRAV2*‐silenced leaves displayed reduced staining (Figure S1). These results indicate that SlRAV2 may promote a transient acceleration of viral replication in infiltrated leaves, thereby raising the ROS levels in the cells, accelerating cell necrosis, and inhibiting virus spread from the infiltrated leaves to the systemic leaves. This result is similar to the one published by Sun and Folimonova (2019) and Sun et al. (2021), who showed that p33 encoded by the citrus tristeza virus (CTV) can be specifically recognised by the plant defence response, increasing the ROS accumulation, and thereby controlling virus accumulation and spread. These findings show that the strategy for inhibiting systemic viral infection mentioned in this article is not uncommon.

As an antiviral TF, RAV may be subject to viral attack or may defend against viral infection. For instance, PbRAV1 from pear interacts with the RSS of ASGV, thereby enhancing antiviral defence (Xie et al. 2024). Similarly, VvRAV1 from grapevine activates the SA‐mediated antivirus signalling (Zhang et al. 2022), while OsRAV15 in rice activates the JA‐mediated antiviral immunity (Zhang, Huang, et al. 2025). In this study, we found an interaction between V2 and SlRAV2 through Y2H screening of a tomato cDNA library, and subsequent validation using Y2H, pull‐down, and BiFC experiments confirmed this interaction (Figure 1A–C). Further truncation analysis showed that the B3 domain (204‐306 aa) of SlRAV2 was sufficient for the interaction with V2 in yeast (Figure S4), it revealed that B3 is the key functional domain for the SlRAV2/V2 interaction site. Interestingly, SlRAV2 and V2 seem to interact mainly within the nucleus. Confocal microscopy revealed distinct nuclear colocalisation of the two proteins, with only weak punctate green fluorescence visible in the cytoplasm (Figure 1C). Therefore, based on their interaction, we hypothesised that the subcellular localisation of both proteins might be affected—a prediction subsequently validated by our experimental results (Figure 5A). Consistent with previous reports in rice (Guo et al. 2024), SlRAV2 localised exclusively to the nucleus in plants, while V2 exhibited both nuclear and cytoplasmic distribution, with forming small aggregates in the endoplasmic reticulum—also aligning with earlier studies (Zhang et al. 2012; Zhao et al. 2020; Zhao, Zhou, et al. 2021; Zhao et al. 2023; Hak et al. 2015; Moshe et al. 2015). The modulation of SlRAV2's subcellular localisation by V2 represents a key regulatory node in their interaction. While V2 did not alter its own nuclear‐cytoplasmic distribution, it significantly restricted SlRAV2's nuclear accumulation, leading to their co‐presence in both compartments (Figure 5B). This effect is mechanistically linked to V2's interference with SlRAV2's interaction with Importin α (Figure S3)—a critical nuclear import mediator (Xiao et al. 2025)—thereby partially sequestering SlRAV2 in the cytoplasm. This finding contrasts with previous reports that V2 facilitates the nuclear entry of certain proteins (Zhao et al. 2020, 2023), highlighting the functional role of V2 in modulating nuclear transport mechanisms. Therefore, V2 affects SlRAV2 localisation, and the functional interaction between V2 and SlRAV2 might be due to the influence of their subcellular localisation.

Plants use Argonaute (AGO) proteins as the main components of RISC, which together with Dicer‐like enzymes, process exogenous viral RNA into siRNAs (Alazem and Lin 2015; Duan et al. 2012). In the presence of RNA‐dependent RNA polymerase, the RNA silencing signal is continuously amplified and transmitted throughout the plant to resist viral infection. Consequently, disruption of siRNA biogenesis or mobility compromises RNA silencing signals. V2 acts as an RSS and binds to ds siRNAs, inhibiting RISC assembly and preventing systemic silencing (Zhang et al. 2012). Here we demonstrated that V2 interacts with the host protein SlRAV2, altering the subcellular localisation of SlRAV2. This interaction represents a mutual regulatory mechanism impacting both viral and host protein functions. Endres et al. (2010) reported that potyvirus HC‐Pro and carmovirus p38 require the ethylene‐inducible host transcription factor RAV2 to block RNA silencing. Xie et al. (2024) and Zhang et al. (2022) also showed that in pear or in grapevine RAV1 enhances the ability of ASGV and GLRaV‐2 RSS to bind to 21‐nt ds siRNAs. Our results are similar to a previous experiment; prokaryotically expressed SlRAV2 enhanced the ability of V2 to bind to 21‐nt ds siRNAs (Figure 3C). The finding indicates that SlRAV2 enhances the silencing suppression activity of V2 by influencing its ability to bind to 21‐nt ds siRNA. Moreover, the full sequence and C‐terminal region of SlRAV2 interacted with V2, thereby influencing its RSS function, but neither SlRAV2 nor SlRAV2^C^ bound to 21‐nt ds siRNA (Figure 3C). These results reveal that SlRAV2's regulatory effect on V2 is mediated through protein–protein interaction rather than direct RNA binding itself. Mechanistically, V2 influenced SlRAV2's nuclear‐cytoplasmic partitioning, partially retaining SlRAV2 in the cytoplasm where V2 resides, thereby increasing local ds siRNA binding and enhancing V2's localised RSS activity. *Agrobacterium*‐mediated transient infection experiments also confirmed that SlRAV2 indeed enhanced the RSS activity of V2 as a result of its subcellular redistribution (Figure 3A). Collectively, integrating the results from Figures 1, 2, 3, SlRAV2 augments V2's RSS efficacy by protein interaction and strengthening its ds siRNA‐binding capacity, leading to a transient increase in TYLCV accumulation and ROS content in infiltrated tissues. Based on our analysis, we propose that the mode of action of SlRAV2 may involve amplifying viral disease symptoms, thereby prematurely and rapidly inducing localised leaf necrosis, which in turn restricts systemic viral spread. We speculate that this mechanism represents an antiviral strategy mediated by RAV proteins.

V2 interacts with SlRAV2 and alters its nuclear localisation, which potentially impacts SlRAV2's transcriptional regulatory function. RAV family proteins are known to act as TFs; for instance, VvRAV1 can regulate *VvPR1* and enhance the SA‐mediated defence response in grapevine (Zhang et al. 2022). Thus, SlRAV2 probably has a regulatory effect on the *SlPR1* gene, a pathogenesis‐related gene activated upon pathogen attack and instrumental in SA‐dependent antiviral defence (Shi et al. 2025; Essa et al. 2025; Zhou et al. 2025). Some reports indicate that plant virus infection elevates *PR* genes expression (Demi̇rel et al. 2025; Zanini et al. 2025; Zhang, Jia, et al. 2025), and we suspect this may be mediated by SlRAV2. Indeed, overexpression of *SlRAV2* resulted in an increase in *SlPR1* expression (Figure 4A); thus, SlRAV2 positively regulates *SlPR1*. Transient expression assays revealed that SlRAV2 directly activated the *SlPR1* promoter, as co‐expression with SlRAV2 significantly elevated GUS activity (Figure 4B). This effect was further enhanced by V2 (Figure 4C), indicating that V2 enhances SlRAV2's regulatory capacity. EMSA confirmed SlRAV2's specific binding to a conserved ‘CACCTG’ motif recognised by the B3 domain (Yamasaki 2004; Kagaya et al. 1999), and V2 strengthened this binding (Figure 4D). This V2‐mediated enhancement contrasts with a report in grapevine, where a viral protein attenuated the DNA‐binding ability of VvRAV1 (Zhang et al. 2022), highlighting species‐specific regulatory outcomes. Together, SlRAV2 not only enhances V2's RSS activity—facilitating transient TYLCV accumulation in infiltrated leaves, but also synergises with V2 to amplify SA‐mediated defence via *SlPR1* activation. This dual effect may trigger elevated ROS production and localised necrotic lesions, thereby restricting systemic viral spread. These two findings provide a relatively complete account of the antiviral role of SlRAV2.

**FIGURE 6:**
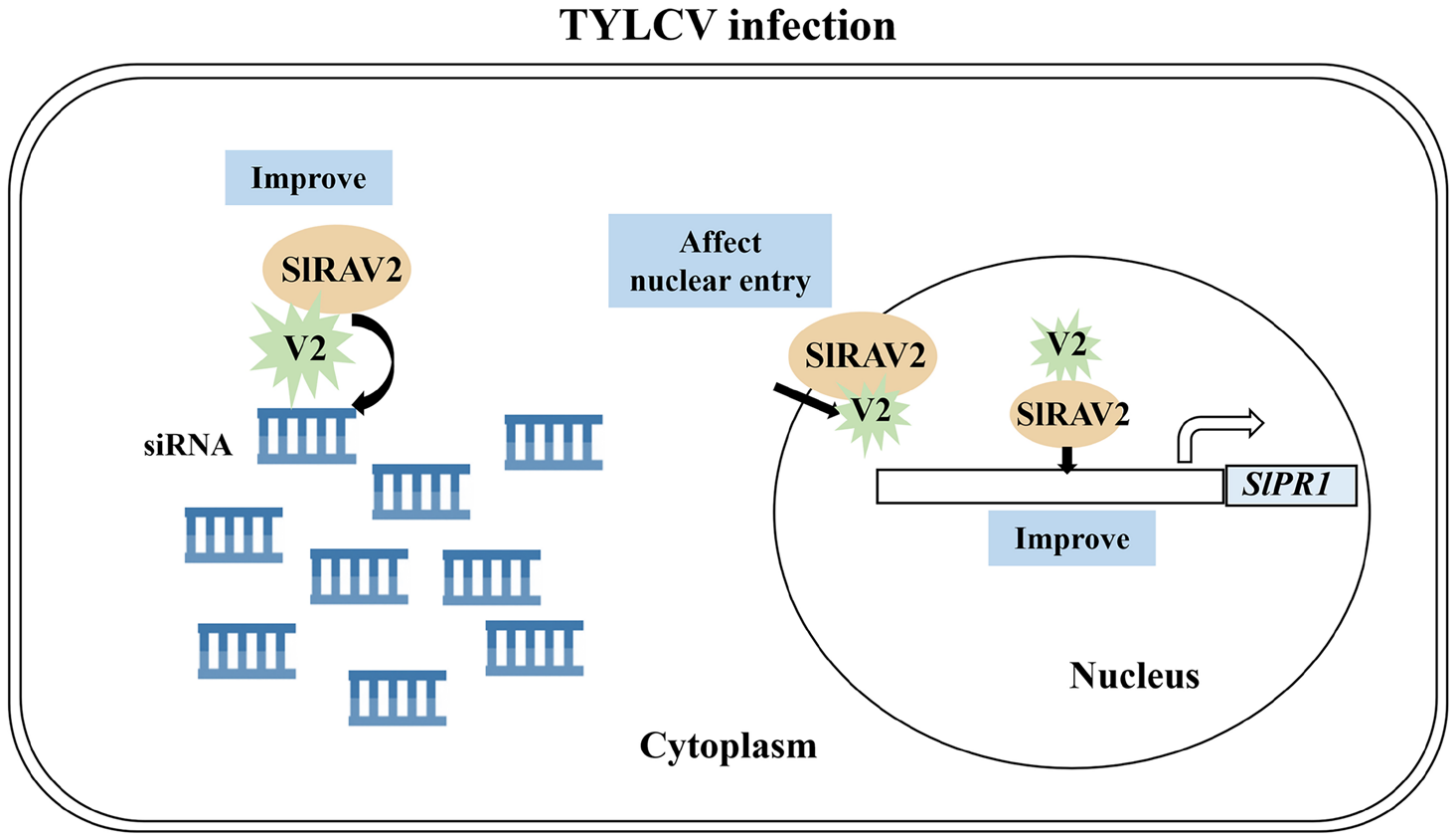
Working model for the SlRAV2/V2 interaction. The interaction between SlRAV2/V2 changes the subcellular localisation of SlRAV2, some SlRAV2 protein enters the cytoplasm and enhances the ability of V2 to bind 21‐nucleotide double‐stranded small interfering RNA (siRNA), causing the accumulation level of tomato yellow leaf curl virus (TYLCV) to surge in the infiltrated leaves. In the nucleus, SlRAV2 utilises V2 to enhance the effect on the *SlPR1* promoter and finally inhibit TYLCV systemic infection.

There is an arms race between viruses and plants. TYLCV avoids excessive cell death to preserve a viable host environment necessary for long‐term infection, actively suppressing plant cell necrotic response (Gorovits et al. 2022). This study demonstrates that SlRAV2 counters this stealth strategy by interacting with RSS V2. Based on our findings, we propose a working model illustrating how SlRAV2 inhibits the systemic infection of TYLCV (Figure 6). In this model, V2 employs SlRAV2 to bind 21‐nt ds siRNA by changing the subcellular localisation of SlRAV2 and effecting the interaction between SlRAV2 and Importin α, thereby augmenting V2's RSS activity. Concurrently, nuclear‐localised V2 potentiates SlRAV2‐mediated transcriptional activation of the *SlPR1p*. This dual modulation transiently elevates local TYLCV accumulation and ROS levels, triggering localised necrosis that restricts viral spread. From a holistic perspective, while it may appear that V2 is exploiting SlRAV2, in reality it is SlRAV2 that is leveraging V2 to mount an antiviral defence. It is noteworthy that although SlRAV2 was demonstrated to restrict systemic viral infection, necrotic symptoms were still observed in systemic tissues, indicating potential limitations of this defence strategy. While V2 modulates SlRAV2 nuclear import and potentially reduces its regulation of *SlPR1*, its cooperative enhancement of SlRAV2 activity predominates, resulting in overall increased regulation of *SlPR1*. We speculate that V2 exerts dual effects on RAV function: it may interfere with certain aspects while partially enhancing others. Consistent with this, V2 does promote a transient rise in viral accumulation; however, this increase is short‐lived, probably due to the counteracting activity of SlRAV2. Eventually, SlRAV2 utilises V2 to achieve the effect of resisting viral infection. The final outcome probably reflects a dynamic balance between viral infection and host defence, which may be influenced by plant developmental stage and environmental conditions. Furthermore, the antiviral function of SlRAV2, particularly its stability, requires further investigation.

## 4 Experimental Procedures

### 4.1 Materials and Methods

Wild‐type *N. benthamiana* plants and tomato seedlings ( *Solanum lycopersicum* ‘Jinpeng No. 1’) with non‐detectable TYLCV were used in this study. The seedlings were grown in individual pots containing commercial soil at 23°C–25°C with a 16‐h light regime, and 4‐week‐old plants were used.

### 4.2 Plasmid Construction

Total RNA was extracted from tomato leaves, followed by reverse transcription to generate complementary DNA (cDNA), which served as the template for PCR amplification of *SlRAV2*. The *V2* gene was amplified from an infectious clone of TYLCV (this plasmid was constructed and stored by our laboratory). *SlRAV2* and *V2* were inserted into the pMD19‐T intermediate vector.

Sequences of *SlRAV2*, *SlRAV2* ^C^, *SlRAV2* ^N^, *SlRAV2* ^B3^ and *V2* were PCR amplified with primer pairs F1/R1, F2/R2, F3/R3, F4/R4 and F5/R5, respectively. PCR products were digested with BamHI/XhoI (for *SlRAV2*, *SlRAV2* ^C^, *SlRAV2* ^N^, *SlRAV2* ^B3^) or EcoRI/SalI (for *V2*) and cloned into pGAD‐T7 and pGBK‐T7.

Then, sequences of *SlRAV2*, *SlRAV2* ^C^, *SlRAV2* ^N^, *V2*, *GFP* and *GUS* were PCR amplified using F6/R6, F7/R7, F8/R8, F9/R9, F10/R10 and F11/R11 primer pairs, respectively. PCR products were digested with XhoI/BamHI (for *SlRAV2*, *SlRAV2* ^C^, and *SlRAV2* ^N^), XhoI/SalI (for *V2*) and XhoI/BamHI (for *GFP* and *GUS*) and cloned into pCambia1300. *V2* was PCR amplified with primer pair F12/R12, and the PCR products were digested with XhoI/SalI and cloned into pCambia1300‐RFP. *SlRAV2* was PCR amplified with primer pair F13/R13, and PCR products were digested with XhoI/BamHI and cloned into pCambia1300‐GFP. *V2* and *GFP* sequences were PCR amplified with F14/R14 and F15/R15 primer pairs, respectively. PCR products were digested with SalI/XhoI (for *V2*) or BamHI/XhoI (for *GFP*) and cloned into pET28a. *SlRAV2* sequences were amplified with primer pair F16/R16, and the PCR products were digested with BamHI/XhoI and inserted into pGEX‐4T‐1 to generate GST‐fusion constructs.

*V2*, *SlRAV2*, *SlRAV2* ^C^ and *SlRAV2* ^N^ sequences were PCR amplified with F17/R17, F18/R18, F19/R19, and F20/R20 primer pairs, respectively. PCR products were digested with BamHI/XhoI (for *SlRAV2*, *SlRAV2* ^C^ and *SlRAV2* ^N^) or SpeI/XhoI (for *V2*) and cloned into BiFC vectors pSPYNE‐35S and pSPYCE‐35S. *SlRAV2* sequences were PCR amplified with primer pair F21/R21. The PCR products were digested with EcoRI/BamHI and cloned into TRV vectors for gene silencing.

Finally, the *SlPR1* promoter region was PCR amplified from tomato leaves using primer pair F22/R22. The PCR products were digested with PstI/SmaI and inserted into pBI121 to produce pBI121‐*SlPR1p*::GUS. All other constructs used in this study were described previously. The primers mentioned above are listed in Table S1.

### 4.3 Y2H Experiment

Co‐transformations were carried out with pairs of plasmids introduced into the yeast strain AH109 via the small‐scale yeast transformation protocol provided by the manufacturer (Coolaber). AH109 was streaked on solid YPDA medium and grown at 30°C until single colonies appeared. One colony was selected and inoculated into broth until the OD_600_ reached 0.3. Yeast cells were centrifuged at 700*g*, washed and resuspended in 1.1 × Tris‐EDTA/lithium acetate buffer. For each reaction, 100 μL competent cells were mixed with 20 μL carrier DNA, 300 μL 50% polyethylene glycol, and the plasmid pair, then incubated at 30°C for 30 min, followed by the addition of 20 μL dimethyl sulppoxide and heat shocked at 42°C for 20 min. After recovery in 1 mL YPD‐Plus at 30°C, cells were resuspended in 0.9% NaCl and plated on the yeast double‐dropout medium (SD/−Trp−Leu) and quadruple‐dropout medium (SD/−Trp−His−Leu−Ade). Then, the yeast strain was cultivated at 30°C for about 3–4 days.

### 4.4 Prokaryotic Expression and Protein Purification

The fusion protein expression vector was transformed into *Escherichia coli* BL21 and cultivated at 37°C. Next, IPTG was added to induce protein expression, followed by using the magnetic beads containing GST‐ or His‐tags, following the manufacturer's instructions (Novagen) to purify the GST‐ or His‐tagged fusion protein (Zhang et al. 2022).

### 4.5 Extraction of Total Protein From Plants and Western Blot Experiment

The protein extraction and western blot experiments were performed using the Fast Western Detection kit (Seven) following the protocol provided in the kit. The film was then photographed to obtain the image.

Protein extraction and western blot analyses were performed as described previously (Li et al. 2018). Dilutions of antibodies or antiserum (noncommercial antibody) were 1:5000 (anti‐GFP, anti‐RFP, anti‐GST, anti‐His and goat anti‐mouse) or 1:2000 (anti‐histone H3, anti‐cFBPase and anti‐MBP). The hybridisation signals were detected using an enhanced chemiluminescence system (eECL Western Blot Kit; ComWin).

### 4.6 *Agrobacterium*‐Mediated Transient Infection

Plasmids pCambia1300‐V2, pCambia1300‐SlRAV2 and pCambia1300‐GFP were transformed into *Agrobacterium tumefaciens* competent cells. Single colonies were selected and cultured at 28°C for 72 h. Bacterial broth cultures were adjusted to OD_600_ of 0.8, harvested by centrifugation at 5000 rpm for 5 min and resuspended in infiltration buffer containing 10 mM MgCl_2_, 10 mM MES and 100 μM acetosyringone. Infiltration was performed by drawing approximately 200–500 μL of the bacterial suspension into a 1 mL syringe and injecting it into the abaxial side of leaves of *N. benthamiana* plants and tomato seedlings.

### 4.7 In‐Vitro Pull‐Down Assay

Fusion proteins were incubated in binding buffer (50 mM Tris–HCl pH 7.5, 100 mM NaCl, 0.25% w/v Triton X‐100, 35‐mM β‐mercaptoethanol) for 2 h at 4°C. Then, 25 μL of His‐Trap (Novagen) was added and the mixture was incubated for an additional 1 h before detecting the pulled‐down proteins using the anti‐GST antibody.

### 4.8 EMSA

The EMSA experiment was conducted using the chemiluminescent EMSA kit (Beyotime). Two biotin‐labelled single‐stranded RNA (sRNA1 and sRNA2) oligonucleotides synthesised by Sangon Biotech were annealed to form 21‐nt ds siRNA probes (sRNA1: 5′‐GUCACUAUGGGUUUAUGG‐3′ and sRNA2: 5′‐CAUAACCAUAGUAGUUGACUG‐3′). Equal amounts (1 ng) of biotin‐labelled DNA or ds siRNA probe were incubated with 2 μg of GST or His‐labelled fusion protein or a mixture of the two proteins. Electrophoresis was performed using a 6% polyacrylamide gel in a buffer (20 mM EDTA, 890 mM Tris‐H_3_BO_3_, pH 8.3). The biotin‐labelled probe was detected using chemiluminescence, and each experiment was repeated three times.

### 4.9 qPCR and RT‐qPCR

qPCR was performed as previously described (Zhang et al. 2022), using the SYBR PrimeScript real‐time PCR Kit (Vazyme), and the cDNA transcribed from RNA as the template. TYLCV genomic DNA was extracted directly using an EZ‐10 Spin Column Plant Genomic DNA Purification Kit (Sangon, Shanghai, China). The following primers, F23/R23, F24/R24, F25/R25 and F26/R26, were used for analysing the relative levels of mRNA (*SlRAV2*, *SlPR1* and *Actin*) and viral DNA. The primers mentioned above are listed in Table S1.

### 4.10 Confocal Microscopy

Fluorescence signals were observed and recorded under a FluoView 3000 confocal microscope (Olympus) equipped with FluoView FV10‐ASW 4.0 Viewer software (Olympus). GFP fluorescence signals were excited at 488 nm, and the signal was captured at 500–540 nm. RFP was excited at 543 nm, and the signal was captured at 570–670 nm. Images were captured using 5% of the maximum light intensity value and a gain of 500–600.

### 4.11 Promoter Transactivation Assay

Recombinant plasmids were introduced into *N. benthamiana* plants by agroinfiltration. The quantitative GUS activity assay was performed as described by Blázquez (2007) using 4‐methylumbelliferyl β‐D‐glucuronide as the substrate.

### 4.12 Nuclear‐Cytoplasmic Fractionation

Infiltrated leaf patches were sampled 3 dpi and pooled. Nuclear‐cytoplasmic fractionation was performed using the Nuclear and Cytoplasmic Protein Extraction Kit (Beyotime) following the manufacturers' protocols.

### 4.13 Statistical and Phylogenetic Analyses

The statistical analyses were performed using SPSS software (SPSS Inc.), and significant differences between treatments were evaluated with Tukey's multiple range test.

## Author Contributions

**Chenwei Zhang:** conceptualization, software, investigation, methodology, data curation, validation, formal analysis, supervision, visualisation, project administration, resource, writing – original draft, writing – review and editing, funding acquisition. **Xin Jia:** conceptualization, software, writing – review and editing, investigation, software, data curation. **Guiyan Fan:** methodology, data curation, software, investigation. **Xiaoli Ren:** methodology, software, data curation, visualisation. **Xing Han:** methodology, data curation, software. **Xiaocong Jiao:** investigation, methodology, validation. **Yuan Cheng:** methodology, software, data curation. **Yajuan Cheng:** data curation, validation, investigation. **Zihan Liu:** data curation, validation, investigation. **Zhehao Yuan:** data curation, validation, investigation. **Yongmin Chen:** conceptualization, resources, project administration, supervision, writing – review and editing.

## Funding

This work was supported by National Natural Science Foundation of China, 32302527.

## Conflicts of Interest

The authors declare no conflicts of interest.

## Supporting information


**Figure S1:** The result of DAB and trypan blue staining in infiltration leaves.

**Figure S2:** Purification of the related prokaryotic expressed protein.

**Figure S3:** V2 affects the interaction between SlRAV2 and SlIMP.

**Figure S4:** The interaction between SlRAV2^B3^ and V2.

**Table S1:** Primer sequences.

## Data Availability

The data that support the findings of this study are available from the corresponding author [Chenwei Zhang], upon reasonable request.

## References

[mpp70230-bib-0001] Alazem, M. , and N. S. Lin . 2015. “Roles of Plant Hormones in the Regulation of Host Virus Interactions.” Molecular Plant Pathology 16: 529–540. 25220680 10.1111/mpp.12204 PMC6638471

[mpp70230-bib-0002] Bar‐Ziv, A. , Y. Levy , V. Citovsky , and Y. Gafni . 2015. “The Tomato Yellow Leaf Curl Virus (TYLCV) V2 Protein Inhibits Enzymatic Activity of the Host Papain‐Like Cysteine Protease CYP1.” Biochemical and Biophysical Research Communications 460, no. 3: 525–529. 25797621 10.1016/j.bbrc.2015.03.063 PMC4439401

[mpp70230-bib-0003] Bar‐Ziv, A. , Y. Levy , H. Hak , et al. 2012. “The Tomato Yellow Leaf Curl Virus (TYLCV) V2 Protein Interacts With the Host Papain‐Like Cysteine Protease CYP1.” Plant Signaling & Behavior 7, no. 8: 983–989. 22827939 10.4161/psb.20935 PMC3474700

[mpp70230-bib-0004] Blázquez, M. 2007. “Quantitative GUS Activity Assay of Plant Extracts.” CSH Protocols 2007: pdb.prot4690. 21357025 10.1101/pdb.prot4690

[mpp70230-bib-0005] Cao, X. , M. Huang , S. Wang , T. Li , and Y. Huang . 2024. “Tomato Yellow Leaf Curl Virus: Characteristics, Influence, and Regulation Mechanism.” Plant Physiology and Biochemistry 213: 108812. 38875781 10.1016/j.plaphy.2024.108812

[mpp70230-bib-0006] Cheng, H. , Q. Wang , Z. Zhang , et al. 2023. “The RAV Transcription Factor TEMPRANILLO1 Involved in Ethylene‐Mediated Delay of Chrysanthemum Flowering.” Plant Journal 116, no. 6: 1652–1666. 10.1111/tpj.16453 37696505

[mpp70230-bib-0007] Demi̇rel, S. , M. Usta , G. Korkmaz , et al. 2025. “Recombinant Tritin Protein Exhibits Antiviral Activity Against Zucchini Yellow Mosaic Virus.” BMC Plant Biology 25, no. 1: 978. 40731260 10.1186/s12870-025-07080-x PMC12305900

[mpp70230-bib-0008] Duan, C. G. , Y. Y. Fang , B. J. Zhou , et al. 2012. “Suppression of *Arabidopsis* ARGONAUTE1‐Mediated Slicing, Transgene‐Induced RNA Silencing, and DNA Methylation by Distinct Domains of the Cucumber Mosaic Virus 2b Protein.” Plant Cell 24, no. 1: 259–274. 22247253 10.1105/tpc.111.092718 PMC3289565

[mpp70230-bib-0009] Endres, M. W. , B. D. Gregory , Z. Gao , et al. 2010. “Two Plant Viral Suppressors of Silencing Require the Ethylene‐Inducible Host Transcription Factor RAV2 to Block RNA Silencing.” PLoS Pathogens 6, no. 1: e1000729. 20084269 10.1371/journal.ppat.1000729 PMC2800190

[mpp70230-bib-0010] Essa, T. A. , N. A. H. Fetyan , T. M. Salem , N. Y. Rebouh , A. Kishk , and M. H. Abdelfattah . 2025. “Stimulation of Resistance Genes and Antioxidant Enzymes in Lettuce by Nano Metal Oxides Against Root Rot Caused by *Rhizoctonia solani* .” PLoS One 20, no. 10: e0334506. 41086184 10.1371/journal.pone.0334506 PMC12520340

[mpp70230-bib-0011] Fu, Y. , Z. Qiao , Y. Zhao , et al. 2025. “Identification of RAV Transcription Factors (B3‐Domain‐Containing) and Functional Analysis of OsRAV2 in Rice Blast and Drought Stress.” Journal of Plant Physiology 314: 154605. 40915024 10.1016/j.jplph.2025.154605

[mpp70230-bib-0012] Fukunaga, R. , and J. A. Doudna . 2009. “dsRNA With 5′ Overhangs Contributes to Endogenous and Antiviral RNA Silencing Pathways in Plants.” EMBO Journal 28, no. 5: 545–555. 19165150 10.1038/emboj.2009.2 PMC2657584

[mpp70230-bib-0013] García, B. , L. Bedoya , J. A. García , et al. 2021. “An Importin‐β‐Like Protein From *Nicotiana benthamiana* Interacts With the RNA Silencing Suppressor P1b of the Cucumber Vein Yellowing Virus, Modulating Its Activity.” Viruses 13, no. 12: 2406. 34960675 10.3390/v13122406 PMC8706682

[mpp70230-bib-0014] Garnelo Gómez, B. , D. Zhang , T. Rosas‐Díaz , Y. Wei , A. P. Macho , and R. Lozano‐Durán . 2019. “The C4 Protein From Tomato Yellow Leaf Curl Virus Can Broadly Interact With Plant Receptor‐Like Kinases.” Viruses 11, no. 11: 1009. 31683645 10.3390/v11111009 PMC6893482

[mpp70230-bib-0015] Glick, E. , A. Zrachya , Y. Levy , et al. 2018. “Interaction With Host SGS3 Is Required for Suppression of RNA Silencing by Tomato Yellow Leaf Curl Virus V2 Protein.” Proceedings of the National Academy of Sciences of the United States of America 105: 157–161. 10.1073/pnas.0709036105 PMC2224178 18165314

[mpp70230-bib-0016] Goldman, V. , and H. Czosnek . 2002. “Whiteflies ( *Bemisia tabaci* ) Issued From Eggs Bombarded With Infectious DNA Clones of Tomato Yellow Leaf Curl Virus From Israel (TYLCV) Are Able to Infect Tomato Plants.” Archives of Virology 147, no. 4: 787–801. 12038688 10.1007/s007050200026

[mpp70230-bib-0017] Gorovits, R. , A. Moshe , L. Amrani , R. Kleinberger , G. Anfoka , and H. Czosnek . 2017. “The Six Tomato Yellow Leaf Curl Virus Genes Expressed Individually in Tomato Induce Different Levels of Plant Stress Response Attenuation.” Cell Stress & Chaperones 22, no. 3: 345–355. 28324352 10.1007/s12192-017-0766-0 PMC5425365

[mpp70230-bib-0018] Gorovits, R. , M. Shteinberg , G. Anfoka , and H. Czosnek . 2022. “Exploiting Virus Infection to Protect Plants From Abiotic Stresses: Tomato Protection by a Begomovirus.” Plants 11, no. 21: 2944. 36365396 10.3390/plants11212944 PMC9657025

[mpp70230-bib-0019] Guo, Y. , L. Liu , X. Shi , P. Yu , C. Zhang , and Q. Liu . 2024. “Overexpression of the RAV Transcription Factor *OsAAT1* Confers Enhanced Arsenic Tolerance by Modulating Auxin Hemostasis in Rice.” Journal of Agricultural and Food Chemistry 72, no. 44: 24576–24586. 39436822 10.1021/acs.jafc.4c04334

[mpp70230-bib-0020] Hak, H. , Y. Levy , S. A. Chandran , et al. 2015. “TYLCV‐Is Movement in Planta Does Not Require V2 Protein.” Virology 477: 56–60. 25644513 10.1016/j.virol.2015.01.007

[mpp70230-bib-0021] Hernández, J. A. , G. Gullner , M. J. Clemente‐Moreno , et al. 2016. “Oxidative Stress and Antioxidative Responses in Plant–Virus Interactions.” Physiological and Molecular Plant Pathology 94: 134–148.

[mpp70230-bib-0022] Hong, J. K. , S. C. Lee , B. K. Hwang , et al. 2005. “Activation of Pepper Basic *PR‐1* Gene Promoter During Defense Signaling to Pathogen, Abiotic and Environmental Stresses.” Gene 356: 169–180. 16005163 10.1016/j.gene.2005.04.030

[mpp70230-bib-0024] Jiang, Y. , W. Zheng , J. Li , et al. 2021. “NbWRKY40 Positively Regulates the Response of *Nicotiana benthamiana* to Tomato Mosaic Virus via Salicylic Acid Signaling.” Frontiers in Plant Science 11: 603518. 33552099 10.3389/fpls.2020.603518 PMC7857026

[mpp70230-bib-0025] Kagaya, Y. , K. Ohmiya , and T. Hattori . 1999. “RAV1, a Novel DNA‐Binding Protein, Binds to Bipartite Recognition Sequence Through Two Distinct DNA‐Binding Domains Uniquely Found in Higher Plants.” Nucleic Acids Research 27: 470–478. 9862967 10.1093/nar/27.2.470 PMC148202

[mpp70230-bib-0026] Kim, N. , J. Kim , B. Bang , et al. 2016. “Comparative Analyses of Tomato Yellow Leaf Curl Virus C4 Protein‐Interacting Host Proteins in Healthy and Infected Tomato Tissues.” Plant Pathology Journal 32, no. 5: 377–387. 27721687 10.5423/PPJ.FT.08.2016.0165 PMC5051556

[mpp70230-bib-0027] Kim, S. Y. , Y. C. Kim , J. H. Lee , et al. 2005. “Identification of a CaRAV1 Possessing an AP2/ERF and B3 DNA‐Binding Domain From Pepper Leaves Infected With *Xanthomonas axonopodis pv. glycines* 8ra by Differential Display.” Biochimica et Biophysica Acta 1729, no. 3: 141–146. 15978683 10.1016/j.bbaexp.2005.04.009

[mpp70230-bib-0028] Király, L. , R. Bacsó , R. Albert , I. Schwarczinger , J. K. Nagy , and A. Künstler . 2025. “Suppressing Symptomless Nonhost Resistance of Barley to *Tobacco mosaic virus* by Short‐Term Heat Stress‐Role of Superoxide in Resistance.” Plants 14, no. 17: 2736. 40941901 10.3390/plants14172736 PMC12430613

[mpp70230-bib-0029] Kozieł, E. , K. Otulak‐Kozieł , and P. Rusin . 2024. “Glutathione the “Master” Antioxidant in the Regulation of Resistant and Susceptible Host–Plant Virus Interaction.” Frontiers in Plant Science 15: 1373801. 38533404 10.3389/fpls.2024.1373801 PMC10963531

[mpp70230-bib-0030] Li, C. W. , R. C. Su , C. P. Cheng , et al. 2011. “Tomato RAV Transcription Factor Is a Pivotal Modulator Involved in the AP2/EREBP‐Mediated Defense Pathway.” Plant Physiology 156: 213–227. 21398258 10.1104/pp.111.174268 PMC3091068

[mpp70230-bib-0031] Li, F. , R. Qiao , X. Yang , et al. 2022. “Occurrence, Distribution, and Management of Tomato Yellow Leaf Curl Virus in China.” Phytopathology Research 4: 28.

[mpp70230-bib-0076] Li, M. , J. Zhang , M. Feng , et al. 2018. “Characterization of silencing suppressor p24 of Grapevine leafroll‐associated virus 2.” Molecular Plant Pathology 19, no. 2: 355–368. 10.1111/mpp.12525. 27997767 PMC6638178

[mpp70230-bib-0032] Li, R. , B. T. Weldegergis , J. Li , et al. 2014. “Virulence Factors of Geminivirus Interact With MYC2 to Subvert Plant Resistance and Promote Vector Performance.” Plant Cell 26: 4991–5008. 25490915 10.1105/tpc.114.133181 PMC4311212

[mpp70230-bib-0034] Licausi, F. , M. Ohme‐Takagi , and P. Perata . 2013. “APETALA2/Ethylene Responsive Factor (AP2/ERF) Transcription Factors: Mediators of Stress Responses and Developmental Programs.” New Phytologist 199, no. 3: 639–649. 24010138 10.1111/nph.12291

[mpp70230-bib-0035] Liu, H. , Z. Chang , S. Zhao , et al. 2023. “Functional Identification of a Novel C7 Protein of Tomato Yellow Leaf Curl Virus.” Virology 585: 117–126. 37331112 10.1016/j.virol.2023.05.011

[mpp70230-bib-0036] Luna, A. P. , G. Morilla , O. Voinnet , and E. R. Bejarano . 2012. “Functional Analysis of Gene‐Silencing Suppressors From Tomato Yellow Leaf Curl Disease Viruses.” Molecular Plant–Microbe Interactions 25, no. 10: 1294–1306. 22712505 10.1094/MPMI-04-12-0094-R

[mpp70230-bib-0037] Luo, W. , K. Wang , J. Luo , et al. 2023. “Limonene Anti‐TMV Activity and Its Mode of Action.” Pesticide Biochemistry and Physiology 194: 105512. 37532363 10.1016/j.pestbp.2023.105512

[mpp70230-bib-0038] Moriones, E. , and J. Navas‐Castillo . 2000. “Tomato Yellow Leaf Curl Virus, an Emerging Virus Complex Causing Epidemics Worldwide.” Virus Research 71: 123–134. 11137167 10.1016/s0168-1702(00)00193-3

[mpp70230-bib-0039] Moshe, A. , E. Belausov , A. Niehl , M. Heinlein , H. Czosnek , and R. Gorovits . 2015. “The Tomato Yellow Leaf Curl Virus V2 Protein Forms Aggregates Depending on the Cytoskeleton Integrity and Binds Viral Genomic DNA.” Scientific Reports 5: 9967. 25940862 10.1038/srep09967 PMC4419519

[mpp70230-bib-0040] Pei, M. , P. Yang , J. Li , et al. 2024. “Comprehensive Analysis of Pepper ( *Capsicum annuum* ) RAV Genes Family and Functional Identification of CaRAV1 Under Chilling Stress.” BMC Genomics 25, no. 1: 731. 39075389 10.1186/s12864-024-10639-x PMC11285464

[mpp70230-bib-0041] Poque, S. , H. W. Wu , C. H. Huang , et al. 2018. “Potyviral Gene‐Silencing Suppressor HC‐Pro Interacts With Salicylic Acid (SA)‐Binding Protein 3 to Weaken SA‐Mediated Defense Responses.” Molecular Plant–Microbe Interactions 31: 86–100. 29090655 10.1094/MPMI-06-17-0128-FI

[mpp70230-bib-0042] Pre, M. , M. Atallah , A. Champion , M. De Vos , C. M. Pieterse , and J. Memelink . 2008. “The AP2/ERF Domain Transcription Factor ORA59 Integrates Jasmonic Acid and Ethylene Signals in Plant Defense.” Plant Physiology 147: 1347–1357. 18467450 10.1104/pp.108.117523 PMC2442530

[mpp70230-bib-0043] Raza, A. , W. Su , A. Gao , et al. 2021. “Catalase (CAT) Gene Family in Rapeseed (*Brassica napus* L.): Genome‐Wide Analysis, Identification, and Expression Pattern in Response to Multiple Hormones and Abiotic Stress Conditions.” International Journal of Molecular Sciences 22: 4281. 33924156 10.3390/ijms22084281 PMC8074368

[mpp70230-bib-0044] Rojas, M. R. , J. Hao , R. Salati , et al. 2001. “Functional Analysis of Proteins Involved in Movement of the Monopartite Begomovirus, Tomato Yellow Leaf Curl Virus.” Virology 291, no. 1: 110–125. 11878881 10.1006/viro.2001.1194

[mpp70230-bib-0045] Shi, J. , L. Li , H. Zhou , et al. 2025. “Chitosan Oligosaccharide Regulates Host Defense in Pepper Plants Against Cucumber Mosaic Virus.” Pesticide Biochemistry and Physiology 215: 106654. 41162044 10.1016/j.pestbp.2025.106654

[mpp70230-bib-0046] Sohn, K. H. , S. C. Lee , H. W. Jung , J. K. Hong , and B. K. Hwang . 2006. “Expression and Functional Roles of the Pepper Pathogen‐Induced Transcription Factor RAV1 in Bacterial Disease Resistance, and Drought and Salt Stress Tolerance.” Plant Molecular Biology 61, no. 6: 897–915. 16927203 10.1007/s11103-006-0057-0

[mpp70230-bib-0047] Su, W. , A. Raza , A. Gao , et al. 2021. “Genome‐Wide Analysis and Expression Profile of Superoxide Dismutase (SOD) Gene Family in Rapeseed (*Brassica napus* L.) Under Different Hormones and Abiotic Stress Conditions.” Antioxidants 10: 1182. 34439430 10.3390/antiox10081182 PMC8389029

[mpp70230-bib-0048] Sun, Y. D. , and S. Y. Folimonova . 2019. “The p33 Protein of Citrus Tristeza Virus Affects Viral Pathogenicity by Modulating a Host Immune Response.” New Phytologist 221, no. 4: 2039–2053. 30220089 10.1111/nph.15482

[mpp70230-bib-0049] Sun, Y. D. , L. Zhang , and S. Y. Folimonova . 2021. “Citrus Miraculin‐Like Protein Hijacks a Viral Movement‐Related p33 Protein and Induces Cellular Oxidative Stress in Defense Against Citrus Tristeza Virus.” Plant Biotechnology Journal 19, no. 5: 977–991. 33283396 10.1111/pbi.13523 PMC8131049

[mpp70230-bib-0050] Tan, H. , X. Zhang , R. Lozano‐Duran , et al. 2024. “Split‐Luciferase Complementation Imaging Assay in Virus–Plant Interactions.” Methods in Molecular Biology 2724: 235–245. 37987910 10.1007/978-1-0716-3485-1_17

[mpp70230-bib-0051] Wang, B. , X. Yang , Y. Wang , Y. Xie , and X. Zhou . 2018. “Tomato Yellow Leaf Curl Virus V2 Interacts With Host Histone Deacetylase 6 to Suppress Methylation‐Mediated Transcriptional Gene Silencing in Plants.” Journal of Virology 92, no. 18: e00036‐18. 29950418 10.1128/JVI.00036-18 PMC6146709

[mpp70230-bib-0052] Wang, L. , P. Fan , T. Jimenez‐Gongora , et al. 2022. “The V2 Protein From the Geminivirus Tomato Yellow Leaf Curl Virus Largely Associates to the Endoplasmic Reticulum and Promotes the Accumulation of the Viral C4 Protein in a Silencing Suppression‐Independent Manner.” Viruses 14, no. 12: 2804. 36560808 10.3390/v14122804 PMC9784378

[mpp70230-bib-0053] Wartig, L. , A. Kheyr‐Pour , E. Noris , et al. 1997. “Genetic Analysis of the Monopartite Tomato Yellow Leaf Curl Geminivirus: Roles of V1, V2, and C2 ORFs in Viral Pathogenesis.” Virology 228, no. 2: 132–140. 9123819 10.1006/viro.1996.8406

[mpp70230-bib-0054] Woo, H. R. , J. Kim , J. Kim , et al. 2010. “The RAV1 Transcription Factor Positively Regulates Leaf Senescence in *Arabidopsis* .” Journal of Experimental Botany 61: 3947–3957. 20826506 10.1093/jxb/erq206 PMC2935868

[mpp70230-bib-0055] Wu, J. B. , F. M. Dai , and X. P. Zhou . 2006. “First Report of Tomato Yellow Leaf Curl Virus in China.” Plant Disease 10: 1359. 10.1094/PD-90-1359C 30780951

[mpp70230-bib-0056] Wu, X. , S. Xu , P. Zhao , et al. 2019. “The *Orthotospovirus* Nonstructural Protein NSs Suppresses Plant MYC‐Regulated Jasmonate Signaling Leading to Enhanced Vector Attraction and Performance.” PLoS Pathogens 15, no. 6: e1007897. 31206553 10.1371/journal.ppat.1007897 PMC6598649

[mpp70230-bib-0057] Xiao, Q. , J. Wang , and G. Chen . 2025. “Self‐Interaction Pattern and Targeted Potential Protein Interaction Networks of *Arabidopsis* CTP: Phosphocholine Cytidylyltransferase 1.” Plant Physiology and Biochemistry 229: 110574. 41045891 10.1016/j.plaphy.2025.110574

[mpp70230-bib-0058] Xie, Y. S. , Q. Zeng , W. T. Huang , et al. 2024. “A Novel RAV Transcription Factor From Pear Interacts With Viral RNA‐Silencing Suppressors to Inhibit Viral Infection.” Plant Journal 120, no. 3: 1079–1093. 10.1111/tpj.17037 39312631

[mpp70230-bib-0059] Xue, L. , F. Xia , W. Song , et al. 2025. “A B3 Domain Transcription Factor NtMAB1 Regulates the Release and Outgrowth of Axillary Buds in Tobacco.” Plant Biotechnology Journal 23: 10.1111. 10.1111/pbi.70313 PMC12665059 40823780

[mpp70230-bib-0060] Yamasaki, K. 2004. “Solution Structure of the B3 DNA Binding Domain of the *Arabidopsis* Cold‐Responsive Transcription Factor RAV1.” Plant Cell 16, no. 12: 3448–3459. 15548737 10.1105/tpc.104.026112 PMC535885

[mpp70230-bib-0061] Yue, H. , G. Chen , Z. Zhang , et al. 2024. “Single‐Cell Transcriptome Landscape Elucidates the Cellular and Developmental Responses to Tomato Chlorosis Virus Infection in Tomato Leaf.” Plant, Cell & Environment 47, no. 7: 2660–2674. 10.1111/pce.14906 38619176

[mpp70230-bib-0062] Zanini, A. A. , M. C. Dominguez , and M. S. Rodríguez . 2025. “Exploring Sugar Allocation and Metabolic Shifts in Cassava Plants Infected With Cassava Common Mosaic Virus (CsCMV) Under Long‐Day Photoperiod: Diel Changes in Source and Sink Leaves.” Journal of Plant Research 138, no. 1: 131–145. 39560817 10.1007/s10265-024-01595-4

[mpp70230-bib-0063] Zhang, C. , X. Jia , X. Han , et al. 2025. “Comparison of Tomato Yellow Leaf Curl Virus‐Induced Gene Expression Pattern in Tomato and Tobacco Plants.” Plant Pathology Journal 41, no. 3: 293–310. 40176574 10.5423/PPJ.OA.12.2024.0191 PMC12146705

[mpp70230-bib-0064] Zhang, C. , X. Wang , H. Li , et al. 2022. “GLRaV‐2 Protein p24 Suppresses Host Defenses by Interaction With a RAV Transcription Factor From Grapevine.” Plant Physiology 189, no. 3: 1848–1865. 35485966 10.1093/plphys/kiac181 PMC9237672

[mpp70230-bib-0065] Zhang, H. , C. Huang , C. Gao , et al. 2025. “Evolutionary‐Distinct Viral Proteins Subvert Rice Broad‐Spectrum Antiviral Immunity Mediated by the RAV15‐MYC2 Module.” Advanced Science 12, no. 12: e2412835. 39903806 10.1002/advs.202412835 PMC11948057

[mpp70230-bib-0066] Zhang, J. , J. Dong , Y. Xu , and J. Wu . 2012. “V2 Protein Encoded by Tomato Yellow Leaf Curl China Virus Is an RNA Silencing Suppressor.” Virus Research 163, no. 1: 51–58. 21893116 10.1016/j.virusres.2011.08.009

[mpp70230-bib-0067] Zhao, C. , X. Liu , Q. Gong , et al. 2021. “Three AP2/ERF Family Members Modulate Flavonoid Synthesis by Regulating Type IV Chalcone Isomerase in Citrus.” Plant Biotechnology Journal 19, no. 4: 671–688. 33089636 10.1111/pbi.13494 PMC8051604

[mpp70230-bib-0068] Zhao, S. , P. Gong , J. Liu , et al. 2023. “ *Geminivirus* C5 Proteins Mediate Formation of Virus Complexes at Plasmodesmata for Viral Intercellular Movement.” Plant Physiology 193, no. 1: 322–338. 37306279 10.1093/plphys/kiad338

[mpp70230-bib-0070] Zhao, W. , Y. Ji , S. Wu , et al. 2018. “Single Amino Acid in V2 Encoded by TYLCV Is Responsible for Its Self‐Interaction, Aggregates and Pathogenicity.” Scientific Reports 8: 3561. 29476063 10.1038/s41598-018-21446-2 PMC5824789

[mpp70230-bib-0071] Zhao, W. , S. Wu , E. Barton , et al. 2020. “Tomato Yellow Leaf Curl Virus V2 Protein Plays a Critical Role in the Nuclear Export of V1 Protein and Viral Systemic Infection.” Frontiers in Microbiology 11: 1243. 32587585 10.3389/fmicb.2020.01243 PMC7297916

[mpp70230-bib-0072] Zhao, W. , Y. Zhou , X. Zhou , et al. 2021. “Host GRXC6 Restricts Tomato Yellow Leaf Curl Virus Infection by Inhibiting the Nuclear Export of the V2 Protein.” PLoS Pathogens 7, no. 8: e1009844. 10.1371/journal.ppat.1009844 PMC8389846 34398921

[mpp70230-bib-0073] Zhou, X. , T. Du , X. Ma , et al. 2025. “Transcription Factor VlPAT2 Enhances the Resistance of Grapevine to *Botrytis cinerea* by Promoting ROS Accumulation.” Plant Cell Reports 44, no. 12: 267. 41207887 10.1007/s00299-025-03633-4

[mpp70230-bib-0074] Zhu, L. , F. Liu , X. Lin , et al. 2025. “Analysis of the AP2/ERF Transcription Factor Family in *Eriobotrya japonica* and Its Role in Exogenous Melatonin‐Mediated Regulation of Salt Stress.” Functional & Integrative Genomics 25, no. 1: 161. 40715545 10.1007/s10142-025-01667-1

[mpp70230-bib-0075] Zrachya, A. , E. Glick , Y. Levy , et al. 2007. “Suppressor of RNA Silencing Encoded by Tomato Yellow Leaf Curl Virus‐Israel.” Virology 358, no. 1: 159–165. 16979684 10.1016/j.virol.2006.08.016

