# Supplementary figures and images for: The Tomato Transcription Factor RAV Affects the Systemic Infection of TYLCV by Interacting With V2
Source: Mol Plant Pathol. 2026 Feb 23;27(2):e70230. doi: 10.1111/mpp.70230 (PMC12929193; doi:10.1111/mpp.70230)

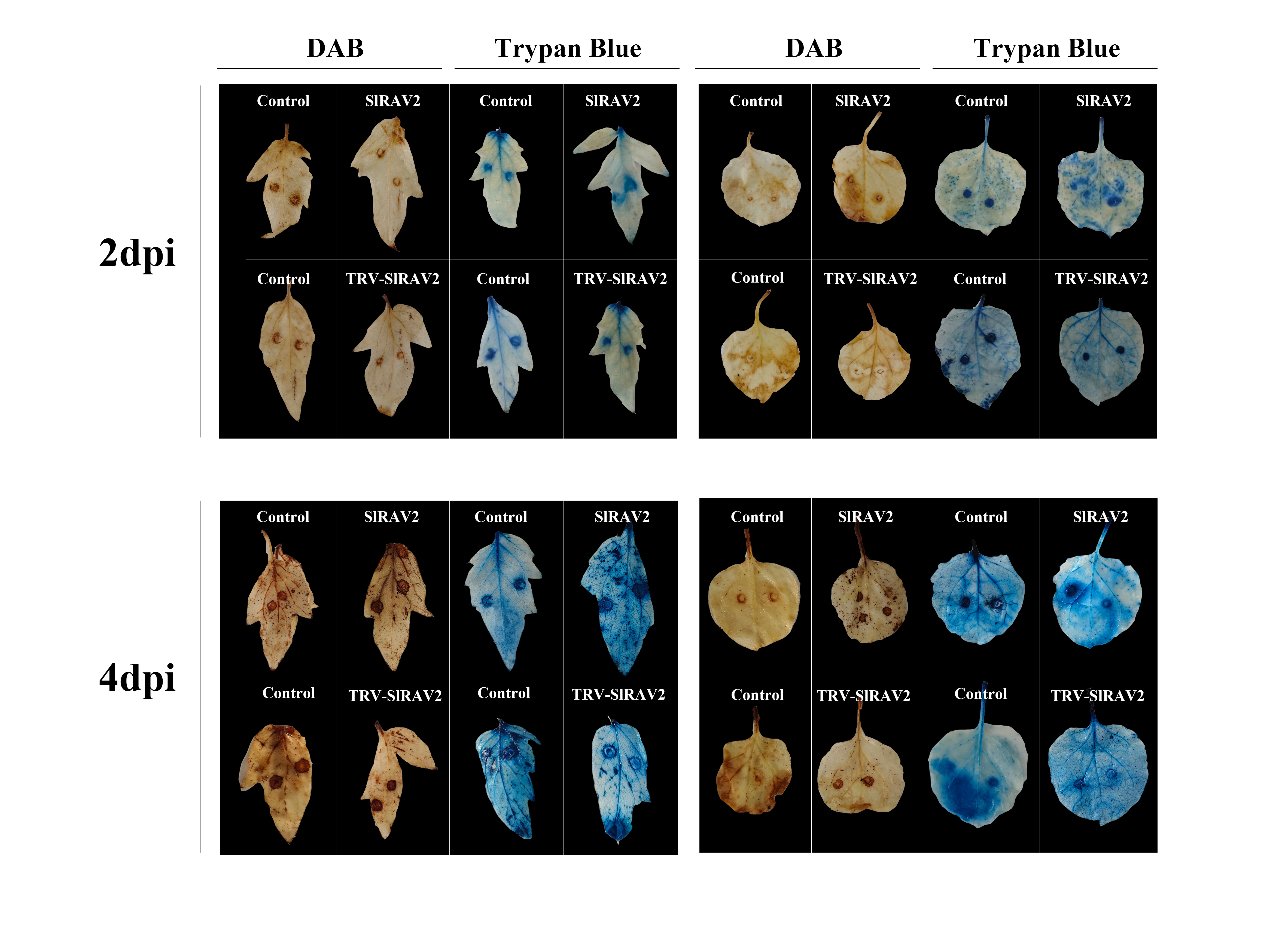

Supplement: Supplementary file 1 — Figure S1: The result of DAB and trypan blue staining in infiltration leaves. [file MPP-27-e70230-s002.jpg]

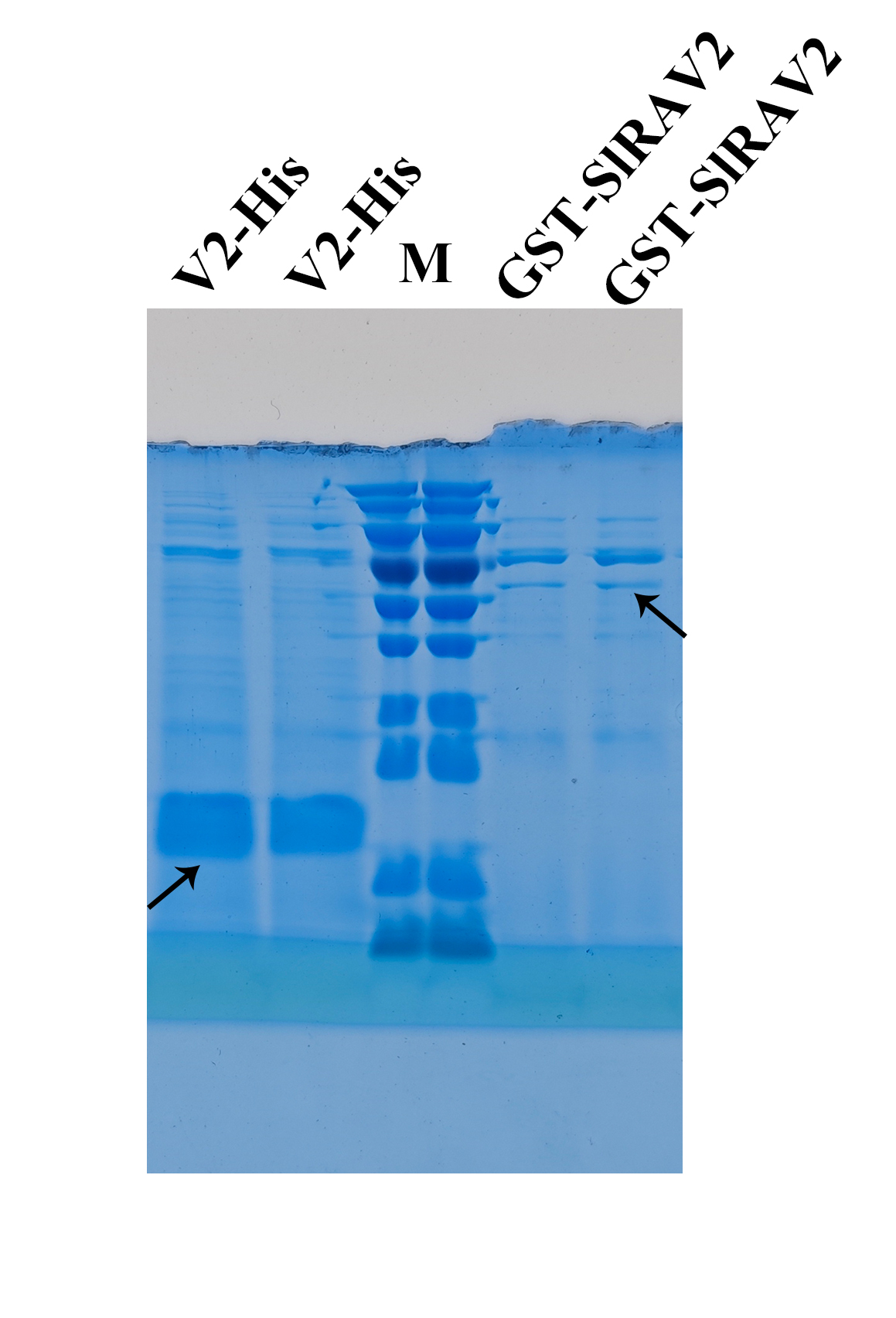

Supplement: Supplementary file 2 — Figure S2: Purification of the related prokaryotic expressed protein. [file MPP-27-e70230-s001.jpg]

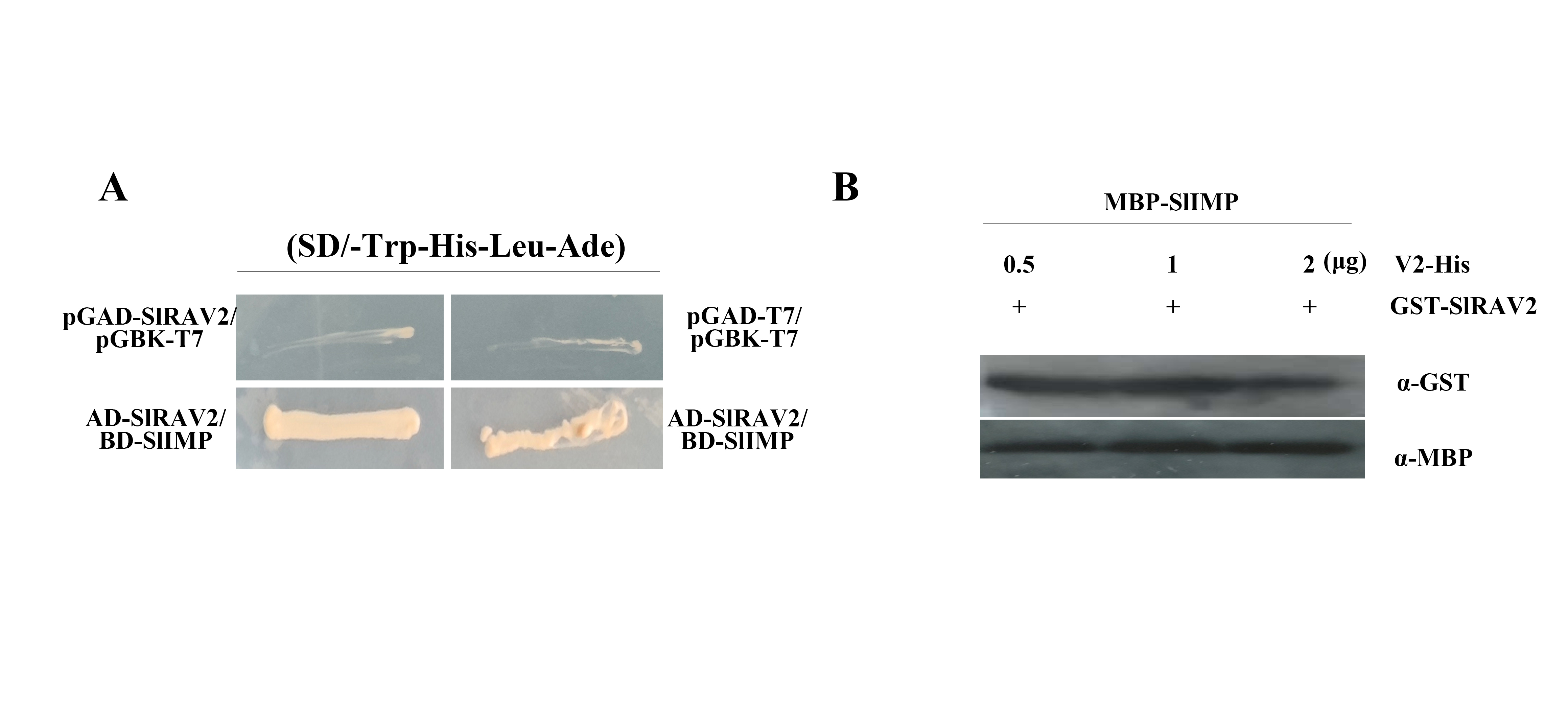

Supplement: Supplementary file 3 — Figure S3: V2 affects the interaction between SlRAV2 and SlIMP. [file MPP-27-e70230-s004.jpg]

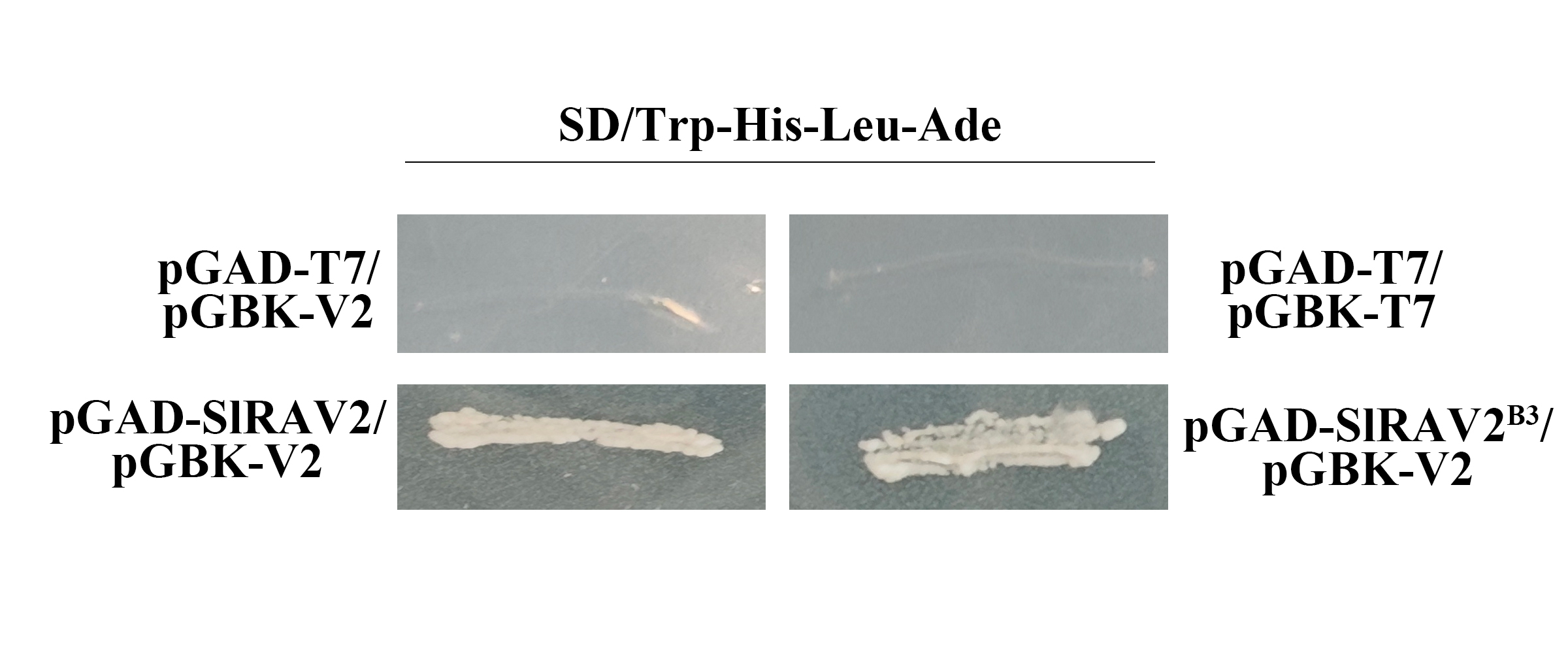

Supplement: Supplementary file 4 — Figure S4: The interaction between SlRAV2B3 and V2. [file MPP-27-e70230-s003.jpg]
