# Supplementary material for: The Tomato Transcription Factor RAV Affects the Systemic Infection of TYLCV by Interacting With V2
Source: Mol Plant Pathol. 2026 Feb 23;27(2):e70230. doi: 10.1111/mpp.70230 (PMC12929193; doi:10.1111/mpp.70230)
Supplement: Supplementary file 5 — Table S1: Primer sequences. [file MPP-27-e70230-s005.docx]

**Table S1: Primer sequences**

| **Vector** | **Primer** | **Sequence** |
| --- | --- | --- |
| pGAD-SlRAV2 | F1 | GCGGATCCATATGGAAGGAAGTATTAGTAGCAT |
| pGAD-SlRAV2 | R1 | ACTCGAGCTTACAAAGCATCAATAATAACCCT |
| pGAD-SlRAV2^C^ | F2 | ATGGATCCATATCGTTGACA TGTTACGTAA |
| pGAD-SlRAV2^C^ | R2 | ACTCGAGCTTACAAAGCATCAATAATAACCCT |
| pGAD-SlRAV2^N^ | F3 | GCGGATCCATATGGAAGGAAGTATTAGTAGCAT |
| pGAD-SlRAV2^N^ | R3 | ATCTCGAGCTTACTCCGCCTTGGAATG |
| pGAD-SlRAV2^B3^ | F4 | ATGGATCCATCTATTTGAAAAAGTTGTTAC |
| pGAD-SlRAV2^B3^ | R4 | ACTCGAGCTTACAAAGCATCAATAATAACC |
| pGBK-V2 | F5 | ATGAATTCATGTGGGATCCACTTCTAAA |
| pGBK-V2 | R5 | ATGTCGACGTCAGGGCTTCGATACAT |
| pCambia1300-SlRAV2 | F6 | ATCTCGAGCTATGGAAGGAAGTATTAGTAGCAT |
| pCambia1300-SlRAV2 | R6 | ATGGATCCTTACAAAGCATCAATAATAACCCT |
| pCambia1300-SlRAV2^C^ | F7 | ATCTCGAGCTATGATCGTTGACATGTTACG |
| pCambia1300-SlRAV2^C^ | R7 | ATGGATCCTTACAAAGCATCAATAATAACCCT |
| pCambia1300-SlRAV2^N^ | F8 | ATCTCGAGCTATGGAAGGAAGTATTAGTAGCAT |
| pCambia1300-SlRAV2^N^ | R8 | ATGGATCCTTACTCCGCCTTG GAATG |
| pCambia1300-V2 | F9 | ATCTCGAGCTATGTGGGATCCACTTCTAAA |
| pCambia1300-V2 | R9 | ATGTCGACTCAGGGCTTCGATACATTC |
| pCambia1300-GFP | F10 | ATCTCGAGCTATGGTGAGCAAGGGCGA |
| pCambia1300-GFP | R10 | GCGGATCCTCAAAGATCTACCAT |
| pCambia1300-GUS | F11 | ATCTCGAGCTATGTTACGTCCTGTAGTTTG |
| pCambia1300-GUS | R11 | ATGGATCCTCATTGTTTGCCTCCC |
| pCambia1300-GFP-SlRAV2 | F12 | ATCTCGAGCTATGGAAGGAAGTATTAGTAGCAT |
| pCambia1300-GFP-SlRAV2 | R12 | ATGGATCCTTACAAAGCATCAATAATAACCCT |
| pCambia1300-RFP-V2 | F13 | ATCTCGAGCTATGTGGGATCCACTTCTAAA |
| pCambia1300-RFP-V2 | R13 | ATGTCGACTCAGGGCTTCGATACATTC |
| pET28a-V2 | F14 | ATGTCGACAA ATGTGGGATCCACTTCTAAA |
| pET28a-V2 | R14 | ATCTCGAGGGGGCTTCGATACAT |
| pET28a-GFP | F15 | ATGGATCCATGGTGAGCAAGGGCGA |
| pET28a-GFP | R15 | ATCTCGAGCTCAAAGATCTACCAT |
| pEGX-4T-1-SlRAV2 | F16 | ATGGATCCATGGAAGGAAGTATTAGTAGCAT |
| pEGX-4T-1-SlRAV2 | R16 | ATCTCGAGCAAAGCATCAATAATAACCCT |
| pSPYCE-35S-V2 | F17 | CGACTAGTATGTGGGATCCACTTCTAAA |
| pSPYCE-35S-V2 | R17 | ATCTCGAGGGGCTTCGATACATTC |
| pSPYNE-35S-SlRAV2 | F18 | ATGGATCCATGGAAGGAAGTATTAGTAGCAT |
| pSPYNE-35S-SlRAV2 | R18 | ATCTCGAGCAAAGCATCAATAATAACCCT |
| pSPYNE-35S-SlRAV2^C^ | F19 | ATGGATCCATGATCGTTGACATGTTACG |
| pSPYNE-35S-SlRAV2^C^ | R19 | ATCTCGAGCAAAGCATCAATAATAACCCT |
| pSPYNE-35S-SlRAV2^N^ | F20 | ATGGATCCATGGAAGGAAGTATTAGTAGC |
| pSPYNE-35S-SlRAV2^N^ | F20 | ATCTCGAGCCTCCGCCTTG GAATG |
| TRV-SlRAV2 | F21 | TGAGTAAGGTTACCGAATTCATGACATCGCGGCCCAGA |
| TRV-SlRAV2 | R21 | GTGAGCTCGGTACCGGATCCTATTAAGTTTCCCAACGT |
| PR1p-F | F22 | ATGTCGACCCGCATAATGTAAGCTCTATATGATAGACT |
| PR1p-R | R22 | ATCCCGGGTATCCCATTATTGGGTTTTGGAG |
| SlRAV2-Q-F | F23 | CTATTTGAAAAAGTTGTTAC |
| SlRAV2-Q-R | R23 | TTACAAAGCATCAATAATAACC |
| SlPR1-Q-F | F24 | ATGGGATACTCCAATATTGC |
| SlPR1-Q-R | R24 | ACATCTTCACAGCACCAGCC |
| ACTIN-Q-F | F25 | GGAAAAGCTTGCCTATGTGG |
| ACTIN-Q-R | R25 | CCTGCAGCTTCCATACC |
| TYLCV-Q-F | F26 | ATGTGGGATCCACTTCTAAA |
| TYLCV-Q-F | R26 | GGGCTTCGATACATTCTG |
| sRNA1 |  | GUCACUAUGGGUUUAUGG |
| sRNA2 |  | CAUAACCAUAGUAGUUGACUG |
| PR1p-probe-1 |  | CTCATAATTCACCTGATTAATCAAT |
| PR1p-probe-2 |  | GAGTATTAAGTGGACTAATTAGTTA |
